# Enhancing Hydrogen Evolution Electrocatalytic Performance in Neutral Media via Nitrogen and Iron Phosphide Interactions

**DOI:** 10.1002/smsc.202100032

**Published:** 2021-05-06

**Authors:** Siyu Zhao, Ruikuan Xie, Liqun Kang, Manni Yang, Xingyu He, Wenyao Li, Ryan Wang, Dan J. L. Brett, Guanjie He, Guoliang Chai, Ivan P. Parkin

**Affiliations:** ^1^ Christopher Ingold Laboratory Department of Chemistry University College London 20 Gordon Street London WC1H 0AJ UK; ^2^ State Key Laboratory of Structural Chemistry Fujian Institute of Research on the Structure of Matter Chinese Academy of Sciences Fuzhou 350002 Fujian P. R. China; ^3^ Department of Chemical Engineering University College London London WC1E 7JE UK; ^4^ Department of Chemical Engineering University of Cincinnati 2600 Clifton Avenue OH 45221 USA; ^5^ School of Chemistry Joseph Banks Laboratories University of Lincoln Green Lane Lincoln LN6 7DL UK; ^6^ Fujian Science & Technology Innovation Laboratory for Optoelectronic Information of China Fuzhou, Fujian 350108 P. R. China; ^7^ Fujian Provincial Key Laboratory of Theoretical and Computational Chemistry Xiamen Fujian 361005 P. R. China

**Keywords:** ex situ characterization, hydrogen evolution reactions, iron phosphide, nitrogen doping, water splitting

## Abstract

It remains a challenge to develop efficient electrocatalysts in neutral media for hydrogen evolution reaction (HER) due to the sluggish kinetics and switch of the rate determining step. Although metal phosphides are widely used HER catalysts, their structural stability is an issue due to oxidization, and the HER performance in neutral media requires improvement. Herein, a new material, i.e., grapevine‐shaped N‐doped iron phosphide on carbon nanotubes, as an efficient HER catalyst in neutral media is developed. The optimized catalyst shows an overpotential of 256 mV at a large current density of 65 mA cm^−2^, which is even 10 mV lower than that of the commercial 20% Pt/C catalyst. The excellent performance of the catalyst is further studied by combined computational and experimental techniques, which proves that the interaction between nitrogen and iron phosphides can provide more efficient active structures and stabilize the metal phosphide electrocatalysts for HER.

## Graphical abstract

N‐doped FeP/carbon nanotube (CNT) grapevine‐shaped catalysts are successfully synthesized by a facile hydrothermal‐annealing method. The introduction of N can enhance the hydrogen evolution performance by optimizing electronic structure and stabilizing the structure of the material simultaneously under working condition. This work provides a facile method to stabilize metal phosphides for electrocatalysis process.

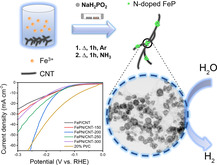

## 1 Introduction

Providing sufficient energy to the growing world population with a minimum impact on the environment requires the development of renewable energy sources.^[^ ^1^, ^2^, ^3^ ^]^ Hydrogen can not only behave as a clean fuel but also as an important raw material for the mass production of various chemicals such as methane and methanol, which make it one of the most promising sustainable energy resources available to replace fossil fuels.^[^ ^4^, ^5^, ^6^ ^]^ A variety of approaches to produce hydrogen, such as electrolysis, photoelectrolysis, and gasification,^[^ ^7^, ^8^, ^9^ ^]^ have been explored over the past decade. Among these methods, the electrolysis of water to produce hydrogen was intensively studied due to its advantages of easy‐to‐obtain reactants, stable high‐purity outputs, and feasible large‐scale production processes.^[^ ^10^, ^11^, ^12^, ^13^ ^]^

The electrolysis of water for the hydrogen evolution reaction (HER) can be performed in acid, neutral, and base media.^[^ ^14^, ^15^, ^16^ ^]^ The HER performance in acid media largely depends on the hydrogen adsorption free energy of the electrocatalysts.^[^ ^17^, ^18^, ^19^ ^]^ The mechanism of HER in acid media is well studied and a volcano plot for metals was achieved for the further optimization of electrocatalysts.^[^ ^1^, ^20^, ^21^ ^]^ However, the mechanism of HER in alkaline and neutral media is more complicated with an additional water dissociation energy barrier slowing down the reaction kinetics, therefore it is worth further investigating the rational design of high‐performance electrocatalysts.^[^ ^22^ ^]^ HER in neutral media is more promising than that in acid or base media due to its inherent advantages including high compatibility, safety, and low cost of electrolytic devices and the potential of direct use for splitting seawater.^[^ ^23^, ^24^ ^]^ However, the application of HER catalysts in neutral media is currently hindered by their relatively lower performance.^[^ ^25^ ^]^ For example, platinum (Pt), one of the most active HER catalysts in acid and alkaline media, shows two to three orders of magnitude lower catalytic activity in neutral media than that in acid media.^[^ ^23^, ^26^ ^]^ Therefore, it is important to provide rational design strategies for high‐performance, low‐cost, and stable electrocatalysts for HER in neutral media.

Recently, transition metal phosphides (TMPs), exhibiting the advantages of low‐cost, excellent electrical conductivity, and high activity, have been explored for HER in neutral media as alternatives to noble metal‐based electrocatalysts.^[^ ^27^, ^28^, ^29^, ^30^, ^31^, ^32^, ^33^, ^34^, ^35^, ^36^, ^37^, ^38^ ^]^ However, the TMPs catalysts usually show an inferior HER performance in neutral media than that in acid and alkaline media, of which the electronic structure can be further optimized by introducing anionic atoms. Meanwhile, the TMPs catalysts show inferior catalytic performance under large current densities compared with commercial Pt/C catalysts, which to date has hindered their potential industrial application. Moreover, recent studies showed that surface reconstruction of metal phosphides during catalytic process can influence the performance of the HER electrocatalysts. Wang et al. developed Fe_0.5_Co_0.5_P nanoparticles supported on mildly oxidized multiwall carbon nanotubes (CNTs) as HER catalysts.^[^ ^31^ ^]^ The X‐ray photoelectron spectroscopy (XPS) results showed the formation of Fe–OH species during HER reaction in 1 m KOH, which is not considered as a good HER catalyst and weakened the catalytic performance. Driess et al. synthesized Ni_12_P_5_ and Ni_2_P as HER catalysts by a facile hydrothermal process.^[^ ^32^ ^]^ The XPS results also showed the surface reconstruction behavior which nickel phosphate species would form on the surface of the electrode during the HER process. Therefore, further development is required for metal phosphide electrocatalysts to stabilize their structure under working conditions and promote their application for HER in neutral media, especially under high current densities.

In this work, we develop a facile strategy to stabilize the structure of metal phosphides during HER and promote intrinsic catalytic activity by introducing N atoms into the structure of the FeP material as HER electrocatalysts for neutral media. Meanwhile, grapevine‐like hollow structures within conductive 3D carbon frameworks were formed to increase active sites. The synthesis–structure–performance relationship was carefully investigated. CNTs can further improve the electric conductivity of the entire structures and disperse FeP active structures evenly on the supported CNT, which are beneficial to the enhanced HER performance. The results showed that materials with nitrogen doped at 200 °C had the lowest overpotential of 158 mV to reach a current density of 10 mA cm^−2^ and possessed a 10 mV lower overpotential than the 20% Pt/C catalyst at a high current density of 65 mA cm^−2^, which proved the potential of these materials for industrial application. The excellent stability of the catalysts was proved by no performance decay after the 20 h stability test. Furthermore, a combined experimental and computational study revealed that the doping of nitrogen can optimize the electronic structure and H* absorption without changing the crystal structure of the material. Compared with pristine FeP, N‐doped FeP (FeP:N) can enhance the efficiency of water dissociation and H_2_ formation confirmed by density functional theory (DFT) calculations. The binding strength between hydroxyl and Fe was greatly weaken in FeP:N which indicates that N dopants can stabilize the structure of FeP by preventing it forming Fe–OH species during HER. The stabilized structure is further proved by ex situ X‐ray fine structure analysis.

## 2 Results and Discussion

**Figure 1:**
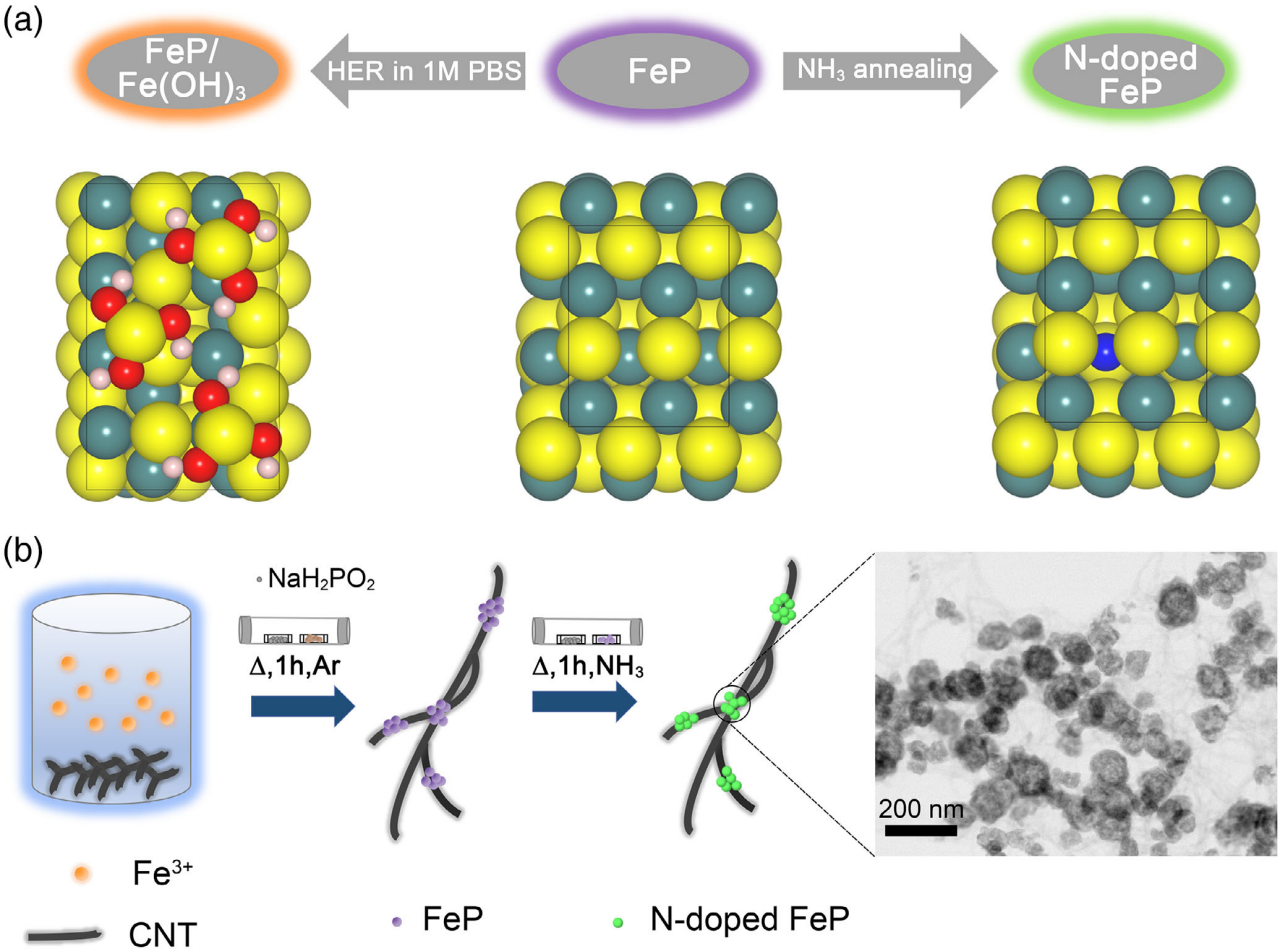
a) Schematic illustration for the surface reconstruction behavior of FeP supported on CNTs under HER condition. (Yellow ball, Fe; red ball, O; pink ball, H; blue ball, N; cyan ball, P). b) Schematic illustration for the preparation of N‐doped FeP catalyst on CNTs. The enlarged graph is the ABF–STEM image of FePN/CNT‐200.

**Figure** **1**a shows the schematic illustration for the surface reconstruction behavior of FeP under HER in 1 m phosphate‐buffered saline (PBS), and Fe(OH)_3_ species would form on the surface of the electrode to hinder the HER performance of the catalysts. The detailed research of the surface reconstruction behavior was discussed in the following ex situ XPS study. In this work, we probe a facile strategy to stabilize the structure of metal phosphides by doping N into the structure of the materials. The N‐doped FeP/CNT materials in this work were synthesized via a three‐step strategy, schematically shown in Figure 1b. The first step involved a facile hydrothermal growth of Fe_2_O_3_ on the mildly oxidized CNTs by using ferric chloride (FeCl_3_) as the Fe source. The ratio of iron source and CNT was optimized, and the morphology of materials were characterized by transmission electron microscopy (TEM). Figure S1a,b, Supporting Information, shows that the diameter of Fe_2_O_3_ nanostructure was smaller than 50 nm and the distribution of materials was uneven when the mass ratio is small, whereas the Fe_2_O_3_ nanostructure would aggregate when the mass ratio was large (Figure S1e,f, Supporting Information), which would decrease the active sites of the catalysts. The material would grow with even distribution on CNT when the optimized mass ratio was applied (Figure S1c,d, Supporting Information). The as‐obtained compounds were transformed into FeP/CNT by annealing Fe_2_O_3_/CNT with sodium dihydrogen phosphate (NaH_2_PO_2_) in a tube furnace at 350 °C for 1 h under an Ar atmosphere. Finally, FeP/CNT was further annealed at 200 °C in a tube furnace for 1 h under NH_3_ atmosphere to obtain N‐doped FeP/CNT (FePN/CNT‐200). Different annealing temperatures between 150 and 300 °C were applied to optimize the performance of the catalysts.

The morphologies of the FePN/CNT‐200 were characterized by annular bright‐field (ABF) and high‐angle annular dark‐field (HAADF) scanning transmission electron microscopy (STEM). Figure 1b shows the catalysts have a grapevine‐like structure with N‐doped FeP nano “grapes” distributed on the CNT “vines.” High‐resolution STEM (Figure S2, Supporting Information) image indicates the d‐spacing of 2.41 nm, corresponding to the (111) plane of FeP. The nano “grapes” were first formed during the hydrothermal process (Figure S1, Supporting Information) and the morphology was not changed during the phosphating process (Figure S3a, Supporting Information). Energy‐dispersive X‐ray spectroscopy (EDS) elemental mapping images of FePN/CNT‐200 (Figure S4, Supporting Information) showed a hollow sphere structure of N‐doped FeP, which may provide abundant active surface areas for the catalysts. However, when the annealing temperature was above 250 °C, the nanostructure was destroyed (Figure S3b, Supporting Information).

**Figure 2:**
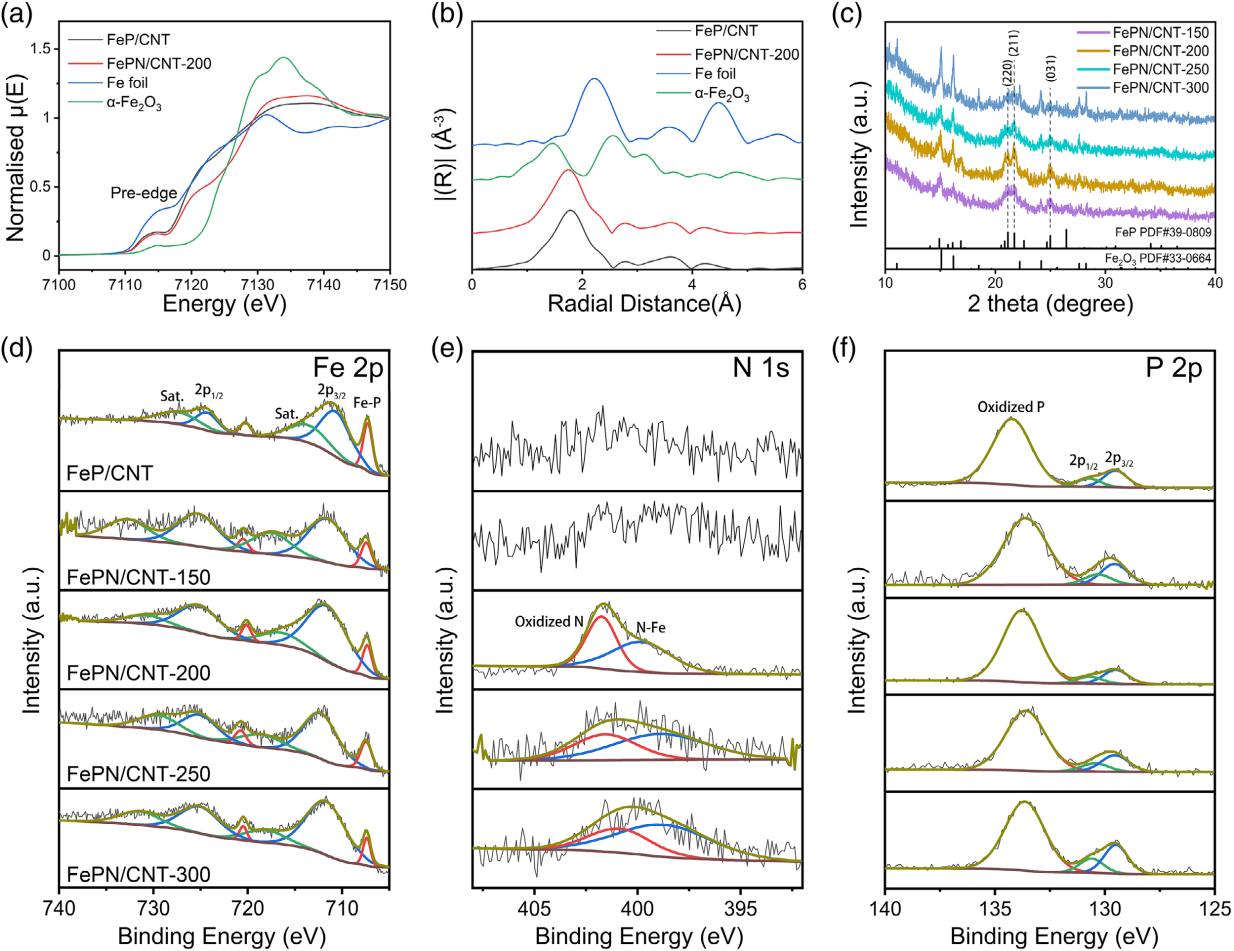
a) Fe K‐edge XANES and b) EXAFS spectra of FeP/CNT, FePN/CNT‐200, Fe foil, and α‐Fe_2_O_3_. c) XRD patterns of FePN/CNT powders prepared at different temperatures. XPS spectra of d) Fe 2p, e) N 1s, and f) P 2p of FePN/CNT prepared at different temperatures.

The composition and elemental valence states of the as‐prepared catalysts were investigated by X‐ray absorption near edge structure (XANES), extended X‐ray absorption fine structure (EXAFS), X‐ray diffraction (XRD), and XPS. **Figure** **2**a shows the XANES results of FeP/CNT. The pre‐edge absorption feature is attributed to 1s → 3 d dipole forbidden transition. The large pre‐edge peak of FeP/CNT is due to the d–p hybridization which provides some electric dipole 1s → 4p character to the transition. Such broad and intense pre‐edge peaks in Fe K‐edge XANES illustrate the metallic Fe nature of the material. In addition, the XANES edge positions of FeP/CNT materials are almost identical to metallic Fe (the first derivative XANES of FeP is also close to metallic Fe). All the aforementioned evidence indicates a metalloid Fe–P alloy property with strong Fe–P interactions (d–p hybridization). On top of this, the further N‐doping into FeP/CNT leads to the formation of FePN/CNT‐200. It is worth mentioning that the pre‐edge feature remains the same after N‐doping, suggesting the same metallic Fe nature of the material. In contrary, the main absorption edge corresponding to the electron transition from 1s to 4p orbitals (which are also hybridized with P 3p orbitals and N 2p orbitals) is significantly modified, indicating the modified electronic structure of valence orbitals by N‐doping. Furthermore, based on the EXAFS results (Figure 2b), the peak of the first shell scattering of FePN/CNT‐200 is shifted to shorter radial distances compared with FeP/CNT due to the contribution from additional Fe–N scattering path, which indicates the FeP structure is maintained but with P substitution by doped N in the first shell. Thus, the N doping does not change the crystal structure but the electronic properties of the material. XRD patterns of the as‐prepared electrodes are shown in Figure 2c and Figure S5, Supporting Information. As shown, XRD peaks of Fe_2_O_3_/CNT and FeP/CNT showed the as‐synthesized materials possessed good crystallization. The XRD peaks of FePN/CNT catalysts at 16.8°, 21.1°, and 21.7° can be indexed to (111), (220), and (211) planes of FeP (PDF No. 39‐0809). The peaks at 24.2°, 27.6°, and 28.2° can be assigned to (116), (214), and (300) planes of Fe_2_O_3_ (PDF No. 33‐0664). The XRD results indicated that the main crystalline structure of FePN/CNT was FeP and the introducing of N kept the original structure unchanged. However, when the annealing temperature was raised above 250 °C, there were more oxidation phases such as Fe_2_O_3_ observed with an increase of annealing temperature, which can be possibly because the samples were prone to oxidation when the crystal structure of the material was destroyed.

The high resolution XPS spectra of Fe 2p, N 1s, and P 2p for N‐doped FeP/CNT materials annealed at different temperatures are shown in Figure 2d–f, and XPS spectra of FeP/CNT are shown in Figure S6, Supporting Information. In the Fe 2p region, two peaks at binding energies of 707.3 and 720.3 eV were assigned to Fe–P species.^[^ ^39^ ^]^ The two peaks at binding energies of 711.8 and 725.3 eV with satellites peaks of 717.5 and 730.5 eV were ascribed to Fe^3+^.^[^ ^40^ ^]^ This can be derived from surface‐oxidized Fe_2_O_3_, which correlated with the XRD results. In the N 1s region, there was no N signal for FePN/CNT‐150, which suggested the N doping was unlikely to occur at the low annealing temperature of 150 °C. The peak at 399.6 eV can be assigned to Fe—N bonds,^[^ ^41^ ^]^ whereas the other peak at 401.8 eV can be indexed to oxidized nitrogen cations.^[^ ^42^ ^]^ The high‐resolution P 2p spectra showed binding energies at 129.5 and 130.5 eV which were assigned to P^3−^ 2p3/2 and 2p1/2 of Fe—P bonds, respectively, whereas the other peak positioned at 133.7 eV can be indexed to surface‐oxidized P species.^[^ ^43^ ^]^ Table S1, Supporting Information, shows the surface quantitative XPS analysis of ratio of Fe:P:N for FePN/CNT‐200, FePN/CNT‐250, and FePN/CNT‐300, which demonstrated a relative approximate ratio of different elements.

**Figure 3:**
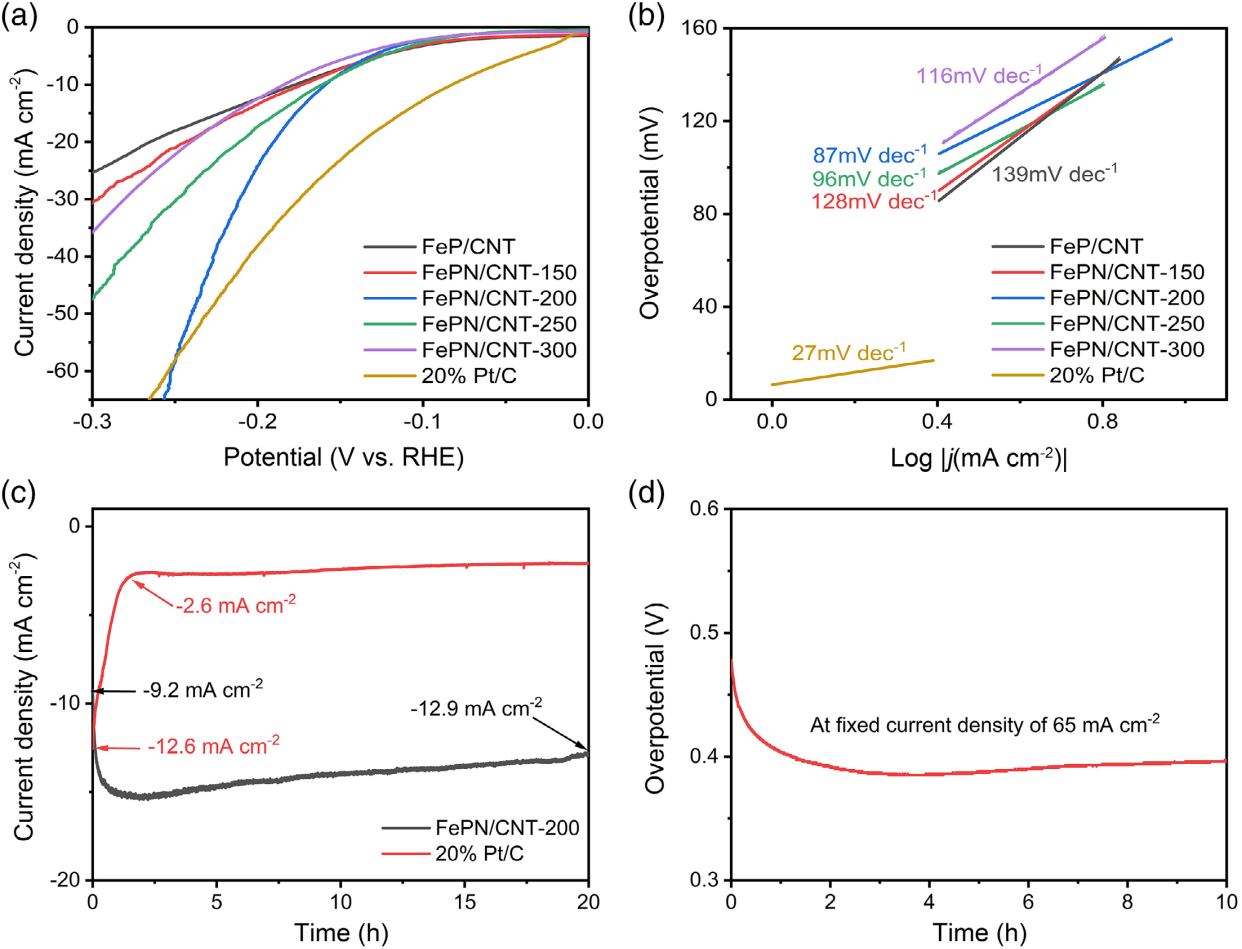
Electrocatalytic measurements of different electrodes for hydrogen evolution in 1 m PBS. a) The polarization curves of different materials. b) Tafel plots derived from the curves in (a). c) Chronoamperometric curves of the FePN/CNT‐200 and 20% Pt/C. d) Chronopotentiometry curve of FePN/CNT‐200 after IR compensation.

The HER electrocatalytic performance of FeP/CNT, FePN/CNT‐150, FePN/CNT‐200, FePN/CNT‐250, FePN/CNT‐300, and 20% Pt/C electrodes were examined in 1 m PBS electrolyte. **Figure** **3**a shows the relevant polarization curves. At a geometric current density of 10 mA cm^−2^, the overpotential for 20% Pt/C is 83 mV, which is better than that of FeP/CNT (178 mV), FePN/CNT‐150 (173 mV), FePN/CNT‐200 (158 mV), FePN/CNT‐250 (161 mV), and FePN/CNT‐300 (182 mV). The results indicate the commercial 20% Pt/C shows a smaller overpotential at low current density whereas FePN/CNT‐200 exhibits the best performance among other materials. However, at a geometric current density of 65 mA cm^−2^, FePN/CNT‐200 shows an overpotential of 256 mV, which is 10 mV lower than that of the commercial 20% Pt/C catalysts (266 mV). This indicates FePN/CNT‐200 catalyst shows better performance than commercial Pt/C catalyst for large‐current commercial hydrogen generation application in neutral media. A comparison of performance for HER electrocatalysts under neutral conditions is shown in Table S2, Supporting Information. To obtain the kinetic information of the as‐prepared electrodes, the corresponding Tafel plots are shown in Figure 3b. The FePN/CNT‐200 electrode exhibits a Tafel slope of 87 mV dec^−1^, which suggests the Volmer reaction is also the rate‐determining step in addition to the Heyrovsky reaction (Table S3, Supporting Information).^[^ ^44^ ^]^ The electrochemical impedance spectroscopy (EIS) spectra of FePN/CNT‐200 (Figure S7, Supporting Information) show a small charge transfer resistance, which indicate high electronic conductivity and charge‐transfer capability.^[^ ^45^ ^]^ Furthermore, a stability test was performed on FePN/CNT‐200 at a fixed potential of −200 mV versus reversible hydrogen electrode (RHE). The test showed that the current density of FePN/CNT‐200 increased by 40% from −9.2 to −12.9 mA cm^−2^ after 20 h. An activation period can be witnessed for 1 h, which can be due to exposing of more effective active sites whereas the surface‐oxidized species dissolved in the electrolyte. The current density was increased to −15.2 mA cm^−2^. After that, the current density was relatively stable and slightly decreased to −12.9 mA cm^−2^, which indicates excellent stability of the catalyst. The 20% Pt/C electrode was performed at a fixed potential of −100 mV versus RHE. Although the current density was −12.6 mA cm^−2^ at the beginning, it dropped fast and kept at around −2.6 mA cm^−2^ which proved the poor stability of the 20% Pt/C electrode. The polarization curves of 20% Pt/C and FePN/CNT‐200 without *iR* correction are shown in Figure S8, Supporting Information. Furthermore, the FePN/CNT‐200 electrode tests were performed with a high fixed current density of 65 mA cm^−2^ for 10 h, and the result showed a similar activation period. The overpotential of FePN/CNT‐200 electrode started from −470 mV and kept at around ‐395 mV, which proved the excellent stability of the electrode even under a large current density. To better understand the remarkable catalytic property of FePN/CNT‐200, the electrochemically active surface area (ECSA) was investigated using a typical cyclic voltammetry (CV) method (Figure S9, Supporting Information). The double‐layer capacitance (*C*_dl_) of FePN/CNT‐200 is 14.5 mF cm^−2^ whereas the *C*_dl_ of FePN/CNT‐150, FePN/CNT‐250, and FePN/CNT‐300 is 20.4 mF cm^−2^, 16.7, and 5.7 mF cm^−2^, respectively. The excellent HER performance of FePN/CNT‐200 was further shown by its higher current density at a lower potential and relatively low *C*_dl_ value compared to other electrocatalysts, which proved a higher intrinsic HER activity of FePN/CNT‐200 in the neutral media. Figure S10, Supporting Information, shows the EIS results of different electrodes recorded in 1 m PBS under open‐circuit condition. The resistance values of FeP/CNT, FePN/CNT‐150, FePN/CNT‐200, FePN/CNT‐250, FePN/CNT‐300, and 20% Pt/C are 3.35, 4.73, 3.62, 4.91, 4.32, and 4.83 Ω, respectively. The FePN/CNT‐200 electrodes showed a small resistance which indicated a fast electron transfer ability. Figure S11, Supporting Information, shows the polarization curves of FeP/NCNT, FePN/NCNT‐200, FeP/CNT, and FePN/CNT‐200, which proved the iron phosphides supported on different type of CNTs could get enhanced HER performance after doping of N.

**Figure 4:**
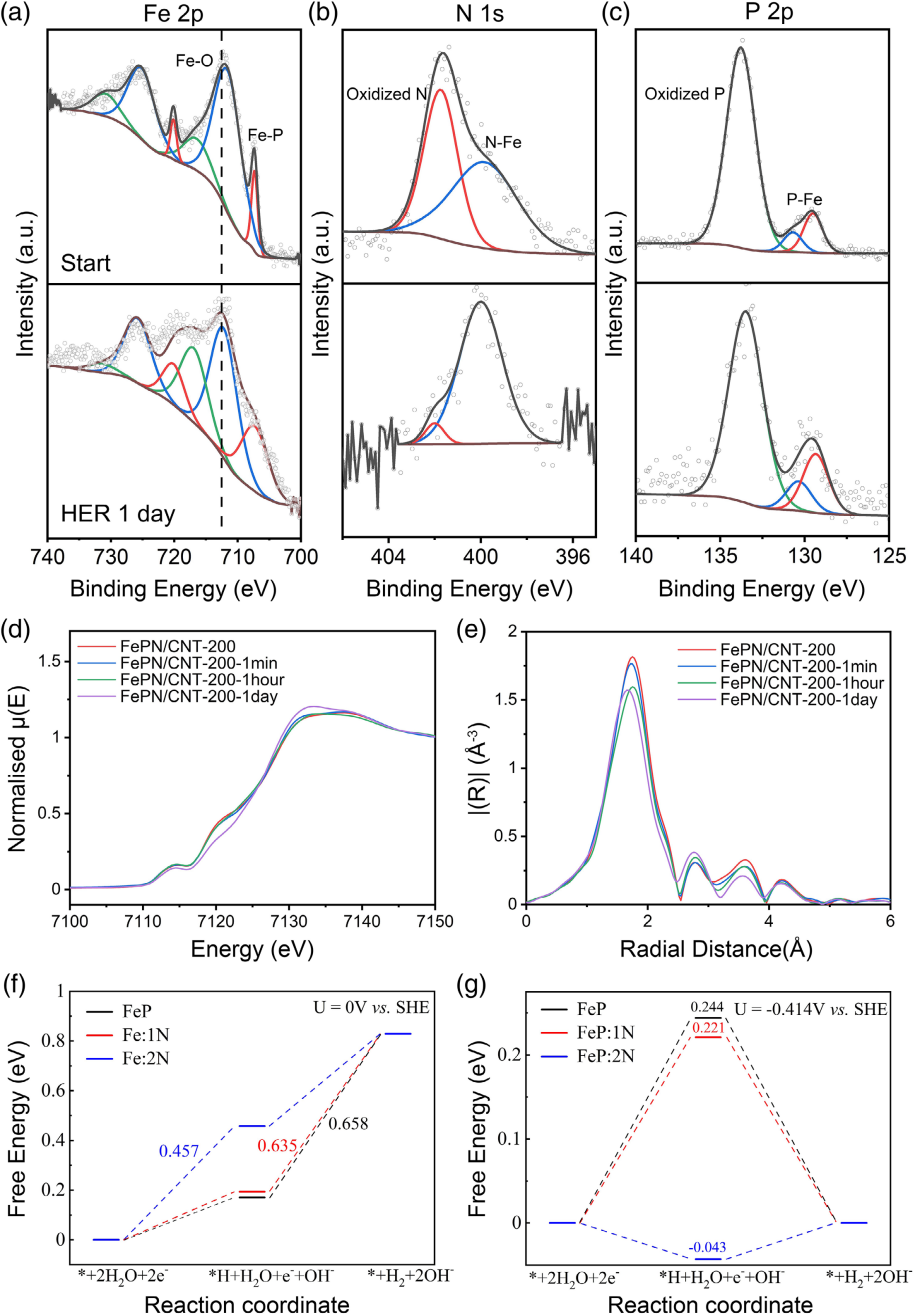
XPS spectra of a) Fe 2p, b) N 1s, and c) P 2p for FePN/CNT‐200 before and after HER test at 10 mA cm^−2^ for 1 day. Ex situ d) XANES and e) EXAFS spectra of FePN/CNT‐200 materials before and after the CA test for 1 min, 1 h, and 1 day. The corresponding EXAFS fitting results are shown in Figure S17 and Table S4, Supporting Information. Free energy diagrams of HER on FeP, Fe:1N, and Fe:2N at f) 0 V versus SHE and g) −0.414 V versus SHE.

To further investigate the mechanism of the catalysts, ex situ XPS tests were performed to study the change of the FeP/CNT and FePN/CNT‐200 before and after the HER test at 10 mA cm^−2^ for 1 day (Figure S12, Supporting Information and **Figure** **4**a–c). The XPS spectra of FeP/CNT showed that the Fe–P peak disappeared after HER test, and there is a shift of Fe–O peak from 711.6 to 712.6 eV, which showed that FeP is not stable during the HER test. For FePN/CNT‐200, the Fe–O peak shifts from 711.6 to 712.5 eV, which can be due to the formation of Fe(OH)_3_ species on the surface of the electrode. A trace of Fe–PO_*x*_ on the surface of the catalysts can be transformed to Fe–OH species during the HER process.^[^ ^31^ ^]^ Based on the previous studies, Fe(OH)_3_ is not considered a good electrocatalysts for HER.^[^ ^46^ ^]^ The formation of iron hydroxide species on the surface of the electrodes can hinder the HER active sites, which can explain the decay of HER performance during stability tests. Figure 4c shows the spectra of P did not change during the HER test for FePN/CNT‐200. Comparing with FeP/CNT, the Fe and P XPS results of FePN/CNT‐200 illustrated that the doping of N can help to stabilize the structure of the catalysts. The oxidized N peak of FePN/CNT‐200 nearly disappeared after the HER test, which can be possibly due to the reduction of the oxidized N in the HER process. The Fe–N peak remains during the HER test, which proves the effectivity and stability of doping with N.

Figure 4d,e show the ex situ XANES and EXAFS spectra of FePN/CNT‐200 electrodes before and after the stability tests for 1 min, 1 h, and 1 day. XANES spectra shown in Figure 4d of these time series show almost no changes for 1 min and 1 h. But for the stability after 1 day, the intensity of the absorption feature (7114 eV) at pre‐edge and main edge (7120 eV) decreased, suggesting that the electrode has been partially oxidized after continues working even at the negative potential. This is in good agreement with EXAFS fitting results (Figure S17 and Table S4, Supporting Information), showing that the coordination number (C.N.) of Fe–O/N increased from 1.0 ± 0.4 to 2.2 ± 0.9, whereas the C.N. of Fe–P dropped from 5.0 ± 0.4 to 3.6 ± 0.8 after 1 day. The absorption feature at 7133 eV indicates the weak presence of Fe(OH)_3_, which corresponds to the XPS results. Thus, the ex situ study indicated the main structure of N‐doped FeP/CNT remained whereas a trace of Fe(OH)_3_ would form during catalytic process, which proved the effectivity of doping with N.

DFT calculations were performed to further investigate how N dopants enhance the HER activity and structural stability. According to the geometry structure of the FeP (110) surface (Figure S13, Supporting Information), seven different H adsorption sites are considered in this article as shown in Figure S14, Supporting Information. The calculated adsorption energies (Figure S15, Supporting Information) indicate that the short Fe–Fe bridge (S–Fe–Fe) site is the most energetically favorable adsorption site for *H intermediate. The two electrochemical elementary steps in neutral condition (pH = 7) and the corresponding free energy diagrams are shown in Figure 4f,g. At 0 V versus standard hydrogen electrode (SHE), the rate‐limiting step is the water dissociation for pristine FeP while the formation of H_2_ for FeP:1 N (FeP doped with one N atom) and FeP:2N (FeP doped with two N atoms). FeP:1N (U_L_ = −0.635 V) shows a more efficient HER than pristine FeP (U_L_ = −0.658 V) and it keeps improving for FeP:2 N (U_L_ = −0.457 V). The equilibrium potential in neutral media is −0.414 V and the free energy diagrams at this potential are shown in Figure 4g. The aforementioned results suggest that doping N into FeP is a promising way to enhance the HER activity in neutral condition.

Previous experimental research revealed a drawback of TMPs as electrocatalysts due to poor stability. Surface deformation and Fe–OH species were observed.^[^ ^31^, ^32^ ^]^ Therefore, understanding the adsorption behavior of hydroxyl on a FeP surface is important to find a way to enhance the stability. As shown in Figure S16, Supporting Information, charge density difference indicates that electrons transfer from Fe to hydroxyl during adsorption process. Therefore, a more positively charged Fe can offer less electrons to hydroxyl and a weaker binding strength should be expected. Since N is more electronegative than P, it will attract more electrons from Fe and leave Fe more positively charged. The Bader effective charges of three samples and corresponding binding energy of hydroxyl are shown in Figure S16b–d, Supporting Information. The negative binding energy becomes positive for FeP:2N, which means the binding strength is very weak and the desorption of hydroxyl will be very energetically favorable. Thus, introducing N can effectively increase the local positive charge on Fe and weaken the Fe—OH bonding, thus preventing the formation of Fe–OH species. Furthermore, the desorption of hydroxyl can offer more active sites for HER.

## 3 Conclusion

In conclusion, a new grapevine‐like structure N‐doped FeP supported on CNT was successfully developed as a HER electrocatalyst by a facile hydro‐thermal‐annealing method. The material has integrated advantages of abundant and efficient active sites provided by the unique structure that promoted intrinsic catalytic activity. The optimized electrocatalyst showed an overpotential of 256 mV to reach a current density of 65 mA cm^−2^, which is 10 mV lower than that of the commercial 20% Pt/C catalyst. The structure of the material was carefully investigated and the change of the material during the HER process was further characterized by ex situ XPS, XANES, and EXAFS. The optimization of the electronic structure and active sites of the FeP electrocatalysts for HER in neutral media by introducing N atoms was proved from both experiments and computational calculations. The transformation to a less active Fe(OH)_3_ was greatly inhibited due to the N‐doping. The DFT calculations predict that N dopants can energetically improve the catalytic activity for HER on FeP and enhance the stability of the crystal structure. Our research offers a new strategy for developing cost‐effective and stable electrocatalysts for HER in neutral media.

## Conflict of Interest

The authors declare no conflict of interest.

## Data Availability Statement

Research data are not shared.

## Supporting information

Supplementary Material
