# Supplementary material for: Enhancing Hydrogen Evolution Electrocatalytic Performance in Neutral Media via Nitrogen and Iron Phosphide Interactions
Source: Small Sci. 2021 May 6;1(7):2100032. doi: 10.1002/smsc.202100032 (PMC11935853; doi:10.1002/smsc.202100032)
Supplement: Supplementary file 1 — Supplementary Material [file SMSC-1-2100032-s001.pdf]

Supporting Information

Enhancing Hydrogen Evolution Electrocatalytic Performance in Neutral Media via Nitrogen and Iron Phosphide Interactions

Siyu Zhao[1]‡, Ruikuan Xie[2]‡, Liqun Kang[3], Manni Yang[1], Xingyu He[5], Wenyao Li[3], Ryan Wang[3], Dan J. L. Brett[3], Guanjie He[1,3,4] Guoliang Chai[2,6,7]* and Ivan P. Parkin[1]**

Experimental section

Material: Sodium hypophosphite monohydrate ($NaH_2PO_2·H_2O$) was purchased from Sigma-Aldrich (UK) Co., Ltd. Iron chloride ($FeCl_3$) was purchased from Sigma-Aldrich (UK) Co., Ltd. Multi-walled carbon nanotube (MWCNT) was purchased from Sigma-Aldrich (UK) Co., Ltd. N, N-Dimethylformamide (DMF) was purchased from Sigma-Aldrich (UK) Co., Ltd. All chemicals were used as received without further purification.

Preparation of N-doped iron phosphide supported on mildly oxidized carbon nanotubes (MOCNT) and N-doped iron phosphide supported on N-doped carbon nanotubes: MOCNT was obtained according to a previous work.[1] In a typical experimental process, 52 mg of $FeCl_3$ was dissolved in 1.6 mL of deionized water and mixed with 8 mg of MOCNT in 16 mL of DMF. Then the mixed solution was stirred and kept at 80 ^oC overnight. Afterwards, the as-prepared solution was transferred to a

20 mL hydrothermal reactor with 6 hours reaction at a temperature of 160 °C. After cooling to room temperature, the product was collected by centrifugation and washed with deionized water and ethanol several times. After being freeze-dried for 1 day, the resulting product was collected and denoted as Fe_2O_3/CNT. The phosphating process was the same with our previous work.[2] Subsequently, 10 mg of Fe_2O_3/CNT was heated in a tube furnace at 350 °C for 1 h with a heating rate of 10 °C/min under an Ar atmosphere to improve the crystallinity. Then 500 mg of $NaH_2PO_2 \cdot H_2O$ was pushed into the tube furnace to heat for one hour at 350 °C under an Ar atmosphere. The waste gas was absorbed by sodium hypochlorite solution. The product was taken out when the tube furnace was cooled down to room temperature and denoted as FeP/CNT. To prepare N-doped FeP/CNT, 10 mg of as-prepared FeP/CNT was annealed in a tube furnace at 200 °C for 1 h with a heating rate of 10 °C/min under NH_3 atmosphere. After cooling to room temperature, the product was taken out and labelled as FePN/CNT-200. Different heating temperatures (150, 250, 300 °C) were applied to investigate the influence of annealing temperature while other conditions were kept the same. The samples obtained from different temperatures were marked as FePN/CNT-150, FePN/CNT-250 and FePN/CNT-300 separately. To make a comparison, the Fe_2O_3 and FeP were prepared without adding MOCNT while other steps were kept the same. N-doped carbon nanotubes were prepared by annealing commercial multiwalled carbon nanotubes under NH_3 flow at 750 °C for 1 h.[1b] The other steps were kept the same as FeP/CNT and FePN/CNT-200 to prepare iron

phosphide prepared on N-doped carbon nanotubes (FeP/NCNT) and N-doped iron phosphide prepared on N-doped carbon nanotubes at 200 ^{o}C (FePN/NCNT-200).

Characterization: The morphology and microstructure of samples was characterized by scanning electron microscope (SEM; Carl Zeiss EVO MA10) and transmission electron microscope (TEM, JEOL, JEM-2100). X-ray diffraction (XRD) patterns were obtained by a STOE SEIFERT diffractometer with detected angular range of $2° < 2\theta < 45°$ with a Mo X-ray radiation source. The chemical states of as-prepared products were detected by X-ray photoelectron spectroscopy (XPS; Thermo scientific K-alpha photoelectron spectrometer). Data processing of XPS results were achieved by CasaXPS with the calibration of adventitious carbon binding energy at 284.6 eV. The mass of the electrodes was weighed accurately by an analytical balance (Ohaus; δ = 0.01 mg). Annular Bright-Field (ABF) and High Angle Annular Dark-Field (HAADF) Scanning Transmission Electron Microscopy (STEM) images were acquired on a probe corrected (CEOS) JEM ARM 200CF electron microscope (JEOL, Japan). The sample was prepared by sprinkling dry sample powder on 400-mesh gold grids with lacey carbon film. The experiment was performed with 200 kV acceleration voltage and a probe current of 76 pA. A 30 μm probe-forming aperture was used, resulting in 23 mrad probe convergence semi-angle. The ABF and HAADF signals were collected simultaneously at 6.0 cm STEM camera length, integrating the scattered electron intensity below 29.3 mrad and between 107.0 to 415.1 mrad, respectively. The regions of interest were exposed to an intense electron beam for 15 min to mitigate the

accumulation of carbon contamination during STEM imaging. Energy-Dispersive X-ray Spectroscopy (EDS) and elemental mapping data were obtained by large solid-angle dual EDS detectors. The EDS spectrum image is 100 × 100 pixels in size with 0.05 second exposure time per pixel. The sample drift was corrected every 30 seconds for EDS mapping. Gatan Microscopy Suite Software was used for both STEM imaging and EDS spectrum imaging data acquisition.

Fe K-edge (7.112 keV) X-ray Absorption Near Edge Structure (XANES) and Extended X-ray Absorption Fine structure (EXAFS) studies were performed at Beamline B18 of the Diamond Light Source (UK).[3,4] Monochromatic X-ray beam was produced by a QEXAFS setup equipped with a fast-scanning Si (111) double crystal monochromator (DCM). A couple of Pt coated harmonic rejection mirrors were inserted between the DCM and ionization chambers to cut off the photons from all higher-order harmonics. The measured energy range was from 6.912 keV to 7.912 keV with 0.25 eV step size. Transmission signals and fluorescence signals were simultaneously acquired by ionization chambers detector and 36-element monolithic Ge detector, respectively. Fe foil was used for energy shift calibration. Powder samples were diluted with cellulose and pressed into 13 mm diameter pellets for measurement in transmission mode. The cycled samples on carbon paper were sealed in Kapton bags and measured in fluorescence mode. The spectra of each sample were measured 10 times and merged to improve the signal-noise ratio. XAFS data was analyzed by Demeter (including Athena and Artemis methods, version 0.9.26).[5] Athena software was used for data extraction and XANES analysis. Artemis software

was used to fit the k^2-weighted EXAFS data. The amplitude reduction factors S_0^2 was calculated to be 0.75 from EXAFS of Fe foil and used as a fixed parameter for EXAFS fitting. The fitting results are shown in Table S4 and Figure S17.

Electrochemical tests: Electrochemical measurements of the as-prepared materials were conducted in a three-electrode cell. 1 M PBS solution was prepared by mixing 1M potassium dihydrogen phosphate (KH_2PO_4) and 1 M dibasic potassium phosphate (K_2HPO_4) and used as the electrolyte. Typically, 2 mg of as-prepared sample was dissolved in a small tube with 190 μL of deionized water, 59 μL of ethanol and 1 μL of 5% Nafion to prepare the ink. Afterwards, the ink was ultrasonicated for 30 min at a temperature lower than 15 ^oC. Then the ink was pasted on 0.5 cm^2 hydrophilic carbon paper (1×2 cm^2) homogeneously and dried in air overnight. The as-prepared carbon papers were used as working electrodes with a loading mass of 4 mg cm^{-2}. A graphite rod was used as the counter electrode and a Ag/AgCl (3M KCl) electrode was used as a reference electrode. The cyclic voltammetry (CV) and linear sweep voltammetry (LSV) measurements were carried out by a Gamry interface 1000 potentiostat. Polarization data were collected at a scan rate of 5 mV s^{-1}. All the potentials were measured against an Ag/AgCl electrode and were converted into the potential vs. the reversible hydrogen electrode (RHE) according to $E_{RHE} = E_{Ag/AgCl} + 0.197 + 0.059pH$. Tafel slopes were determined by fitting the linear regions of the Tafel plots according to the Tafel equation ($\eta = b \log(j) + a$) by replotting the polarization curves. The long-term stability was evaluated by the chronoamperometry

measurement. Electrochemical impedance spectroscopy (EIS) was performed with frequencies from 0.1 to 100,000 Hz with an amplitude of 10 mV. All the LSV measurements are presented with iR compensation.

Computational methods: All the density functional theory (DFT) calculations were performed via the Vienna Ab initio Simulation package (VASP),[6-9] and the projector-augmented plane wave (PAW) pseudopotentials were used for the elements involved.[10] The generalized gradient approximation (GGA) of Perdew, Burke, and Ernzerhof (PBE) was used to treat the exchange-correlation between electrons.[11] The pristine FeP (110) slab calculated in this study is shown in Figure S10 and the bottom two layers kept fixed during the calculation. A vacuum region of greater than 15 Å was added along the direction normal to the slab plane to avoid the interaction between periodic supercells. The electron wave function is expanded in plane waves and a cutoff energy of 500 eV was chosen. The Monkhorst-Pack meshes of (7, 7, 1) and (9, 9, 9) were adopted for the Brillouin zone (BZ) of slabs and primitive cell.[12] The convergence in the energy and force were set to 10^{-4} eV and 0.01 eV/Å, respectively.

The free energies of H_2O (l) and H_2 (g) were used as references when calculating the free energies of reaction intermediates. The adsorption energy for reaction intermediate was calculated as follows.[13]

$$\Delta G = \Delta E_{Total} + \Delta E_{ZEP} - T\Delta S + \Delta G_s - 0.0592*pH - eU \quad (1)$$

where ΔE_{Total} is the calculated adsorption total energy by DFT, ΔE_{ZPE} is zero-point energy, ΔS is entropy, and ΔG_s is solvation energy.[14-16]

For hydrogen evolution reaction (HER) in neutral solution, the elementary steps are:

$* + H_2O + e^- \rightarrow *H + OH^-$ (2)

$*H + H_2O + e^- \rightarrow * + H_2 + OH^-$ (3)

where * denotes a possible reaction site. The calculated HER electrochemical potential can be obtained as follows:

$UL = Mini[-\Delta G_i] / ne$ (4)

where n is the number of electrons transferred for each electrochemical step, and e is the elementary charge. Here, the n is set to 1 for the one-electron transfer step. The meaning of the r.h.s. of the above equation is to select the smallest $[-\Delta G_i]$ among the HER elementary steps.

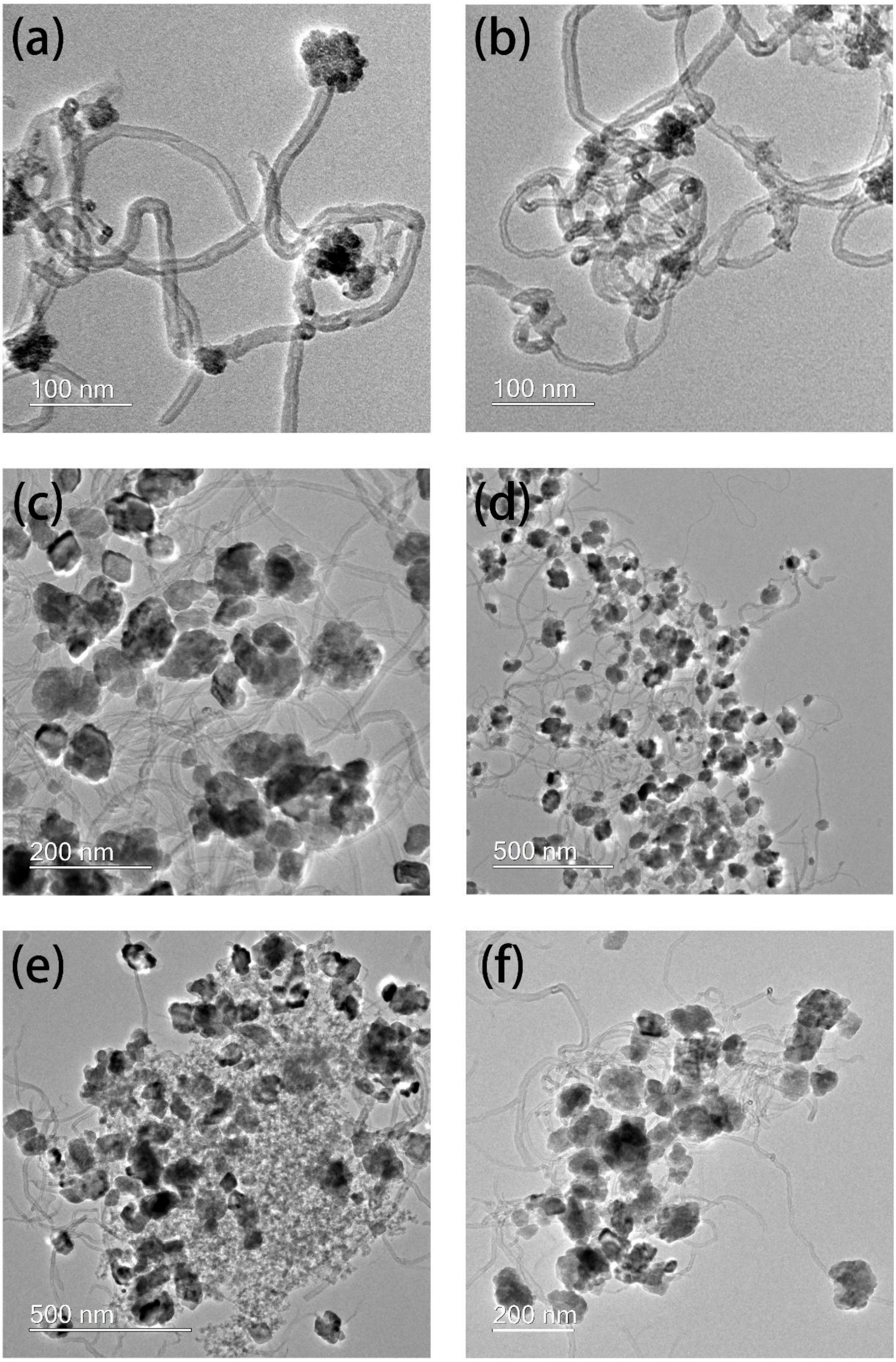

Figure S1. TEM images of Fe_2O_3/CNT prepared with different Fe source and CNT mass ratio (n). (a,b) n = 3.25. (c,d) n=6.5. (e,f) n = 13.

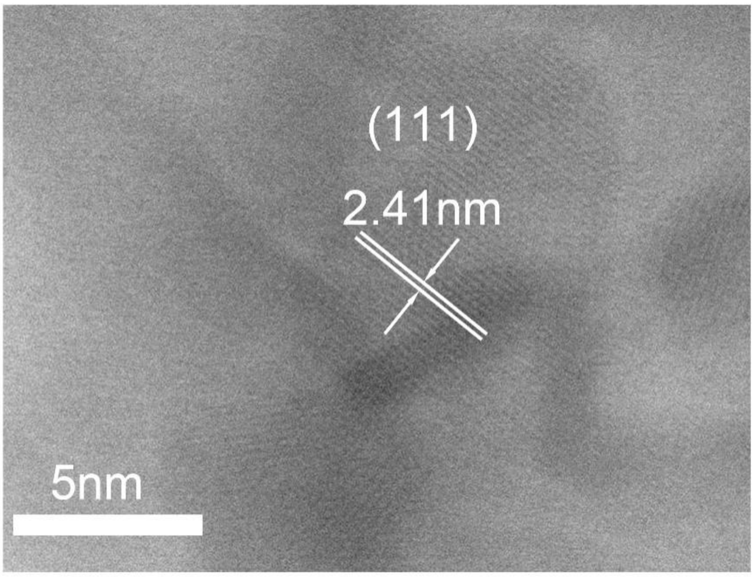

Figure S2. ABF-STEM image of FePN/CNT-200.

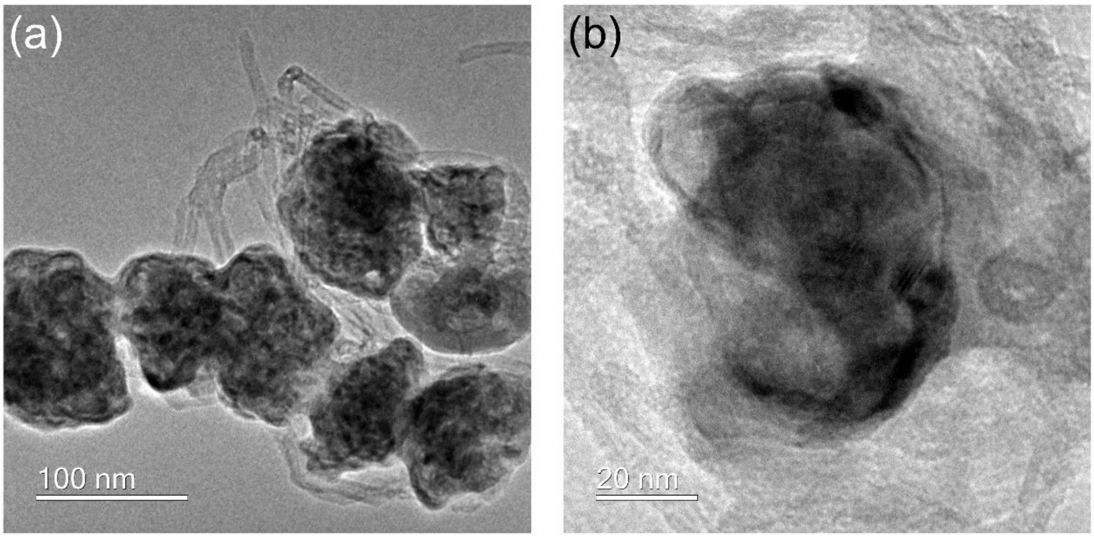

Figure S3. TEM images of (a) FeP/CNT and (b) FePN/CNT-250.

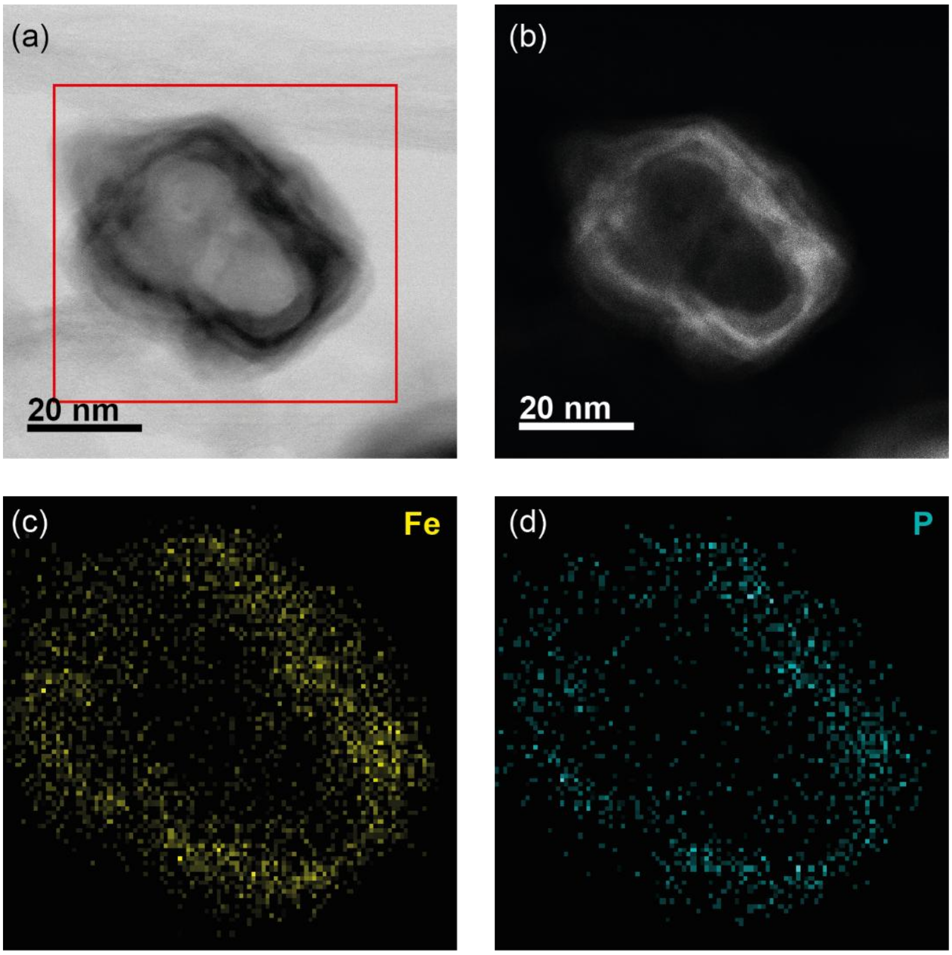

Figure S4. (a) ABF and (b) HAADF-STEM images of FePN/CNT-200. (c-d) Elemental mappings of FePN/CNT-200.

The formation of FeP is based on this reaction: Fe_2O_3 (s) + $2PH_3$ (g) == 2FeP (s) + $3H_2O$ (g). Therefore, there reason for such hollow structure is as follows: First, the volume density of Fe atoms decreased from Fe_2O_3 to FeP while the total amount of Fe is constant. For α-Fe_2O_3 (hematite, space group: $R\bar{3}c$), each crystal cell contains 4 Fe atoms with a cell volume of 100 $Å^3$, which means 25 $Å^3$ per Fe site. While for FeP (space group: *Pbnm*), there are 4 Fe atoms in each crystal cell within a cell volume of 92.9 $Å^3$, corresponding to approximately 23 $Å^3$ per Fe site. Since the phase transformation from Fe_2O_3 to FeP started from the surface, a FeP shell over Fe_2O_3 core was firstly formed during the PH_3 annealing. Followed by the continues Fe_2O_3 to FeP conversion at the interface of Fe_2O_3 (core) /FeP (shell), a hollow structure would eventually form due to the volume shrinkage of the whole solid phase composite. In addition, each PH_3 gas molecule resulted in 1.5 H_2O molecules, leading to a significant volume expansion in the gas phase. Such gas volume expansion happened within the formed FeP shell at the Fe_2O_3 (core) /FeP (shell) interface. Therefore, the formed H_2O gases would 'blow' the FeP shell to an even bigger size. In a brief summary, the volume shrinkage of solid phase and the volume expansion of the gas phase during PH_3 annealing caused such formation of hollow structure.

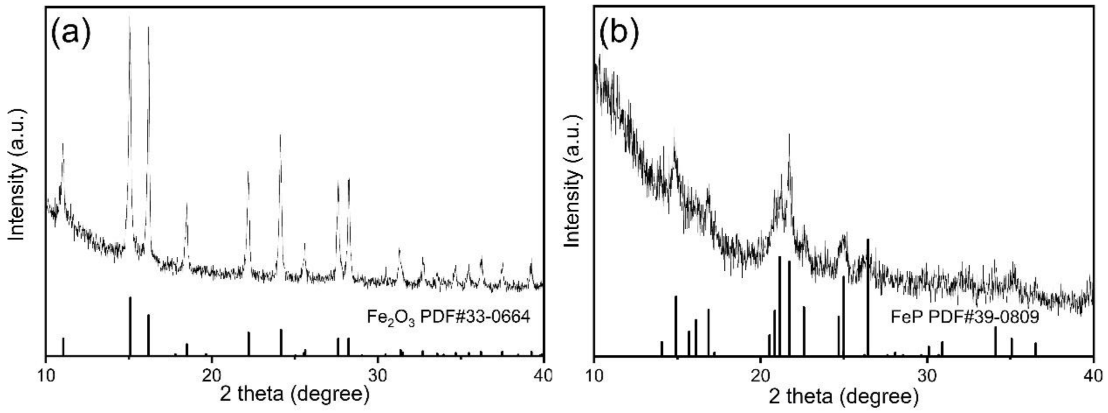

Figure S5. XRD patterns of (a) Fe_2O_3/CNT and (b) FeP/CNT.

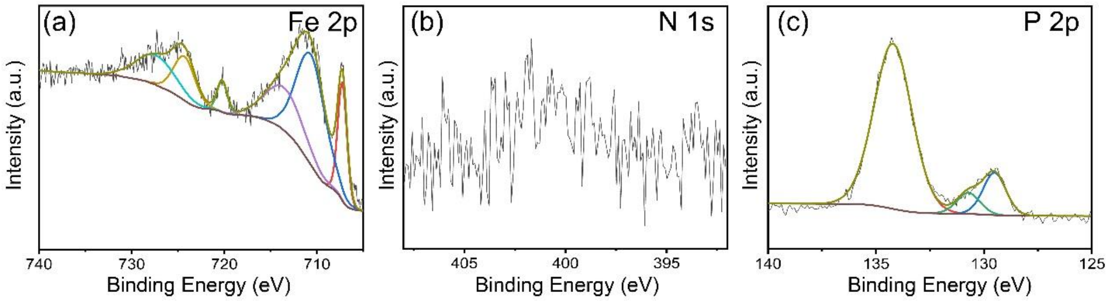

Figure S6. XPS spectra of FeP/CNT.

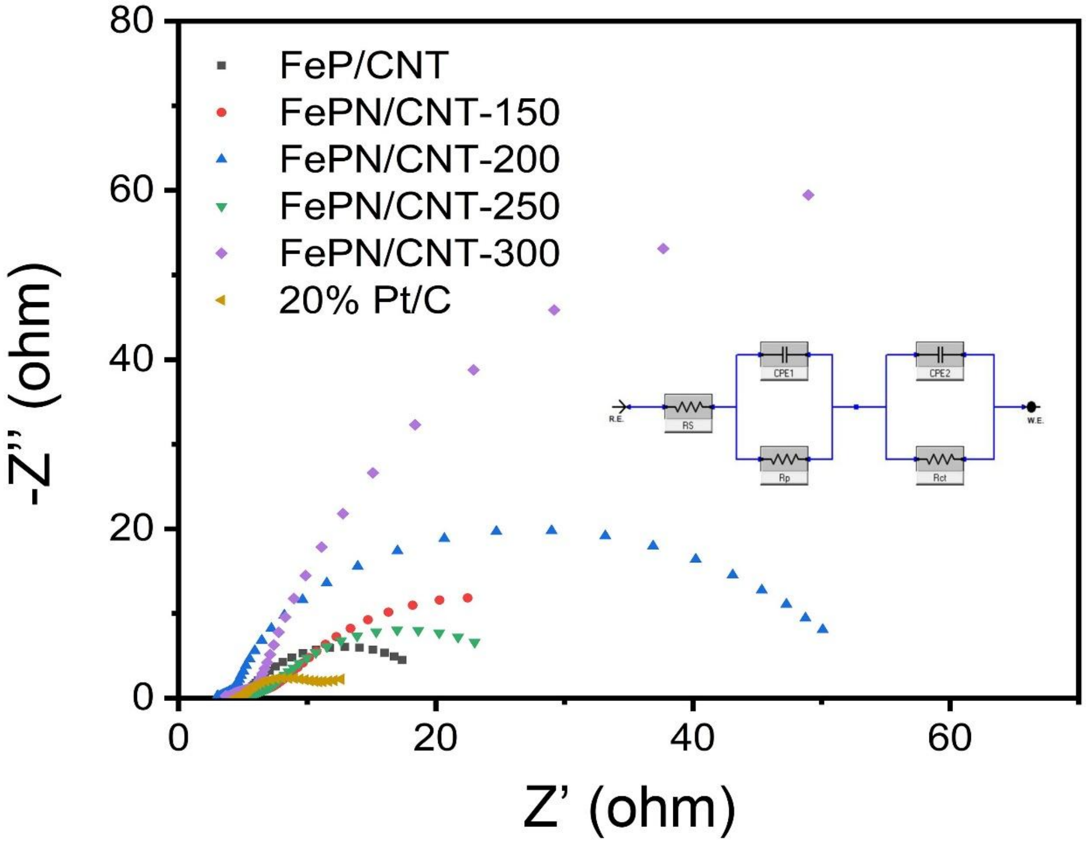

Figure S7. Nyquist plots of electrochemical impedance spectra (EIS) of different electrodes recorded in 1 M PBS; Inset: Two-time-constant model equivalent circuit used for data fitting of EIS spectra. Rs represents the overall series resistance; CPE1 and CPE2 represent the constant phase element and resistance related to the surface porosity; R_p and R_{ct} represent the charge transfer resistance related to the HER process.

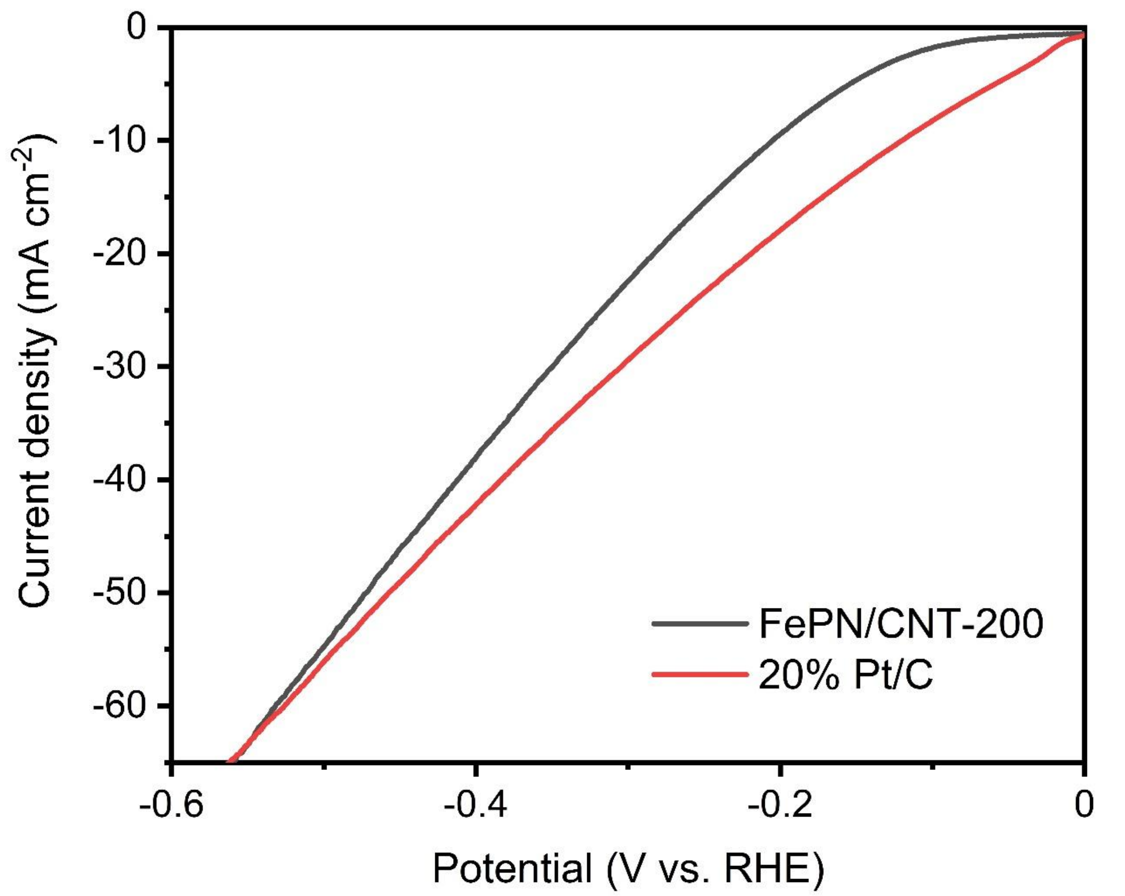

Figure S8. Polarization curves of FePN/CNT-200 and 20% Pt/C without iR compensation in 1M PBS.

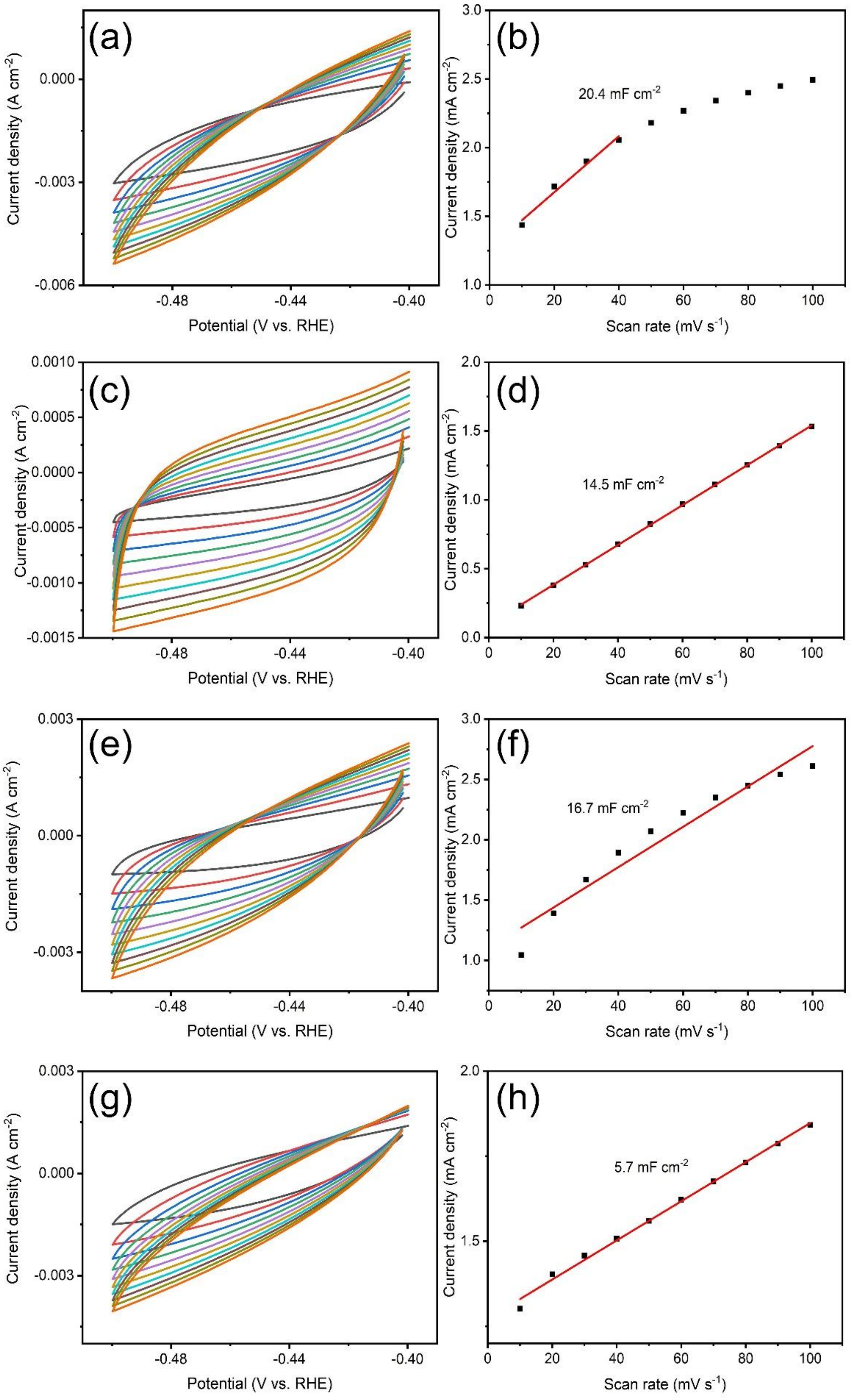

Figure S9. Electrochemical surface area (ECSA) of (a,b) FePN/CNT-150, (c,d) FePN/CNT-200, (e,f) FePN/CNT-250 and (g,h) FePN/CNT-300.

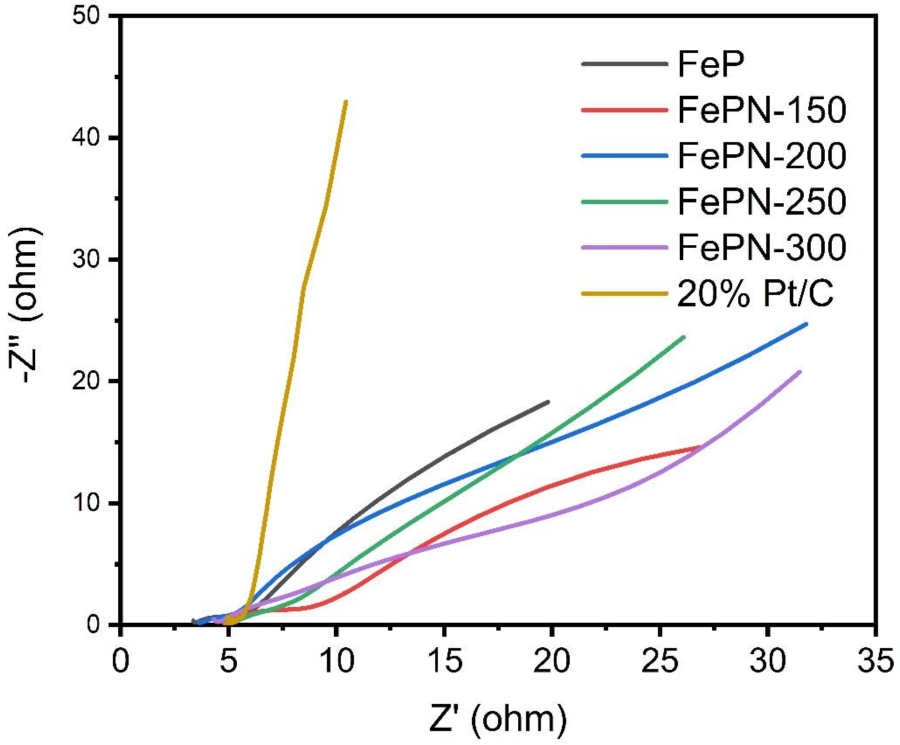

Figure S10. EIS spectra of different electrodes under open circuit potential.

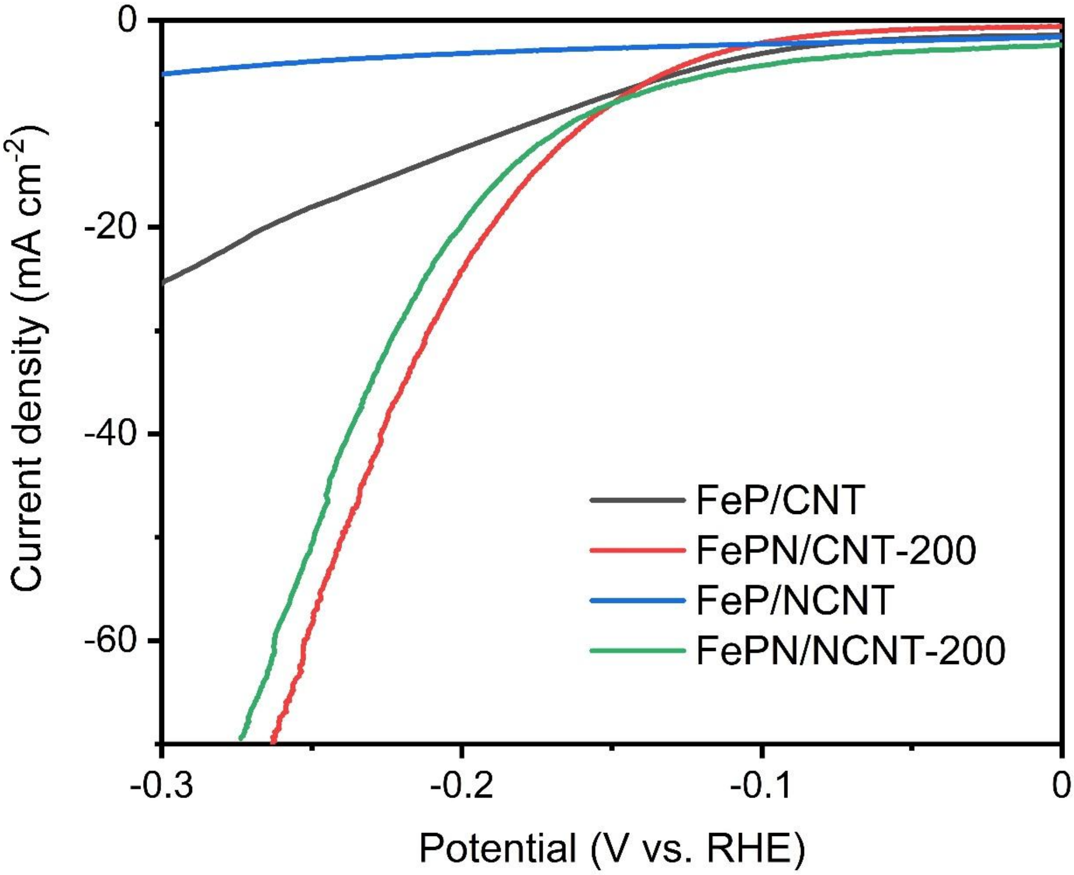

Figure S11. Polarization curves of FeP/CNT, FePN/CNT-200, FeP/NCNT and FePN/NCNT-200 after iR compensation in 1M PBS.

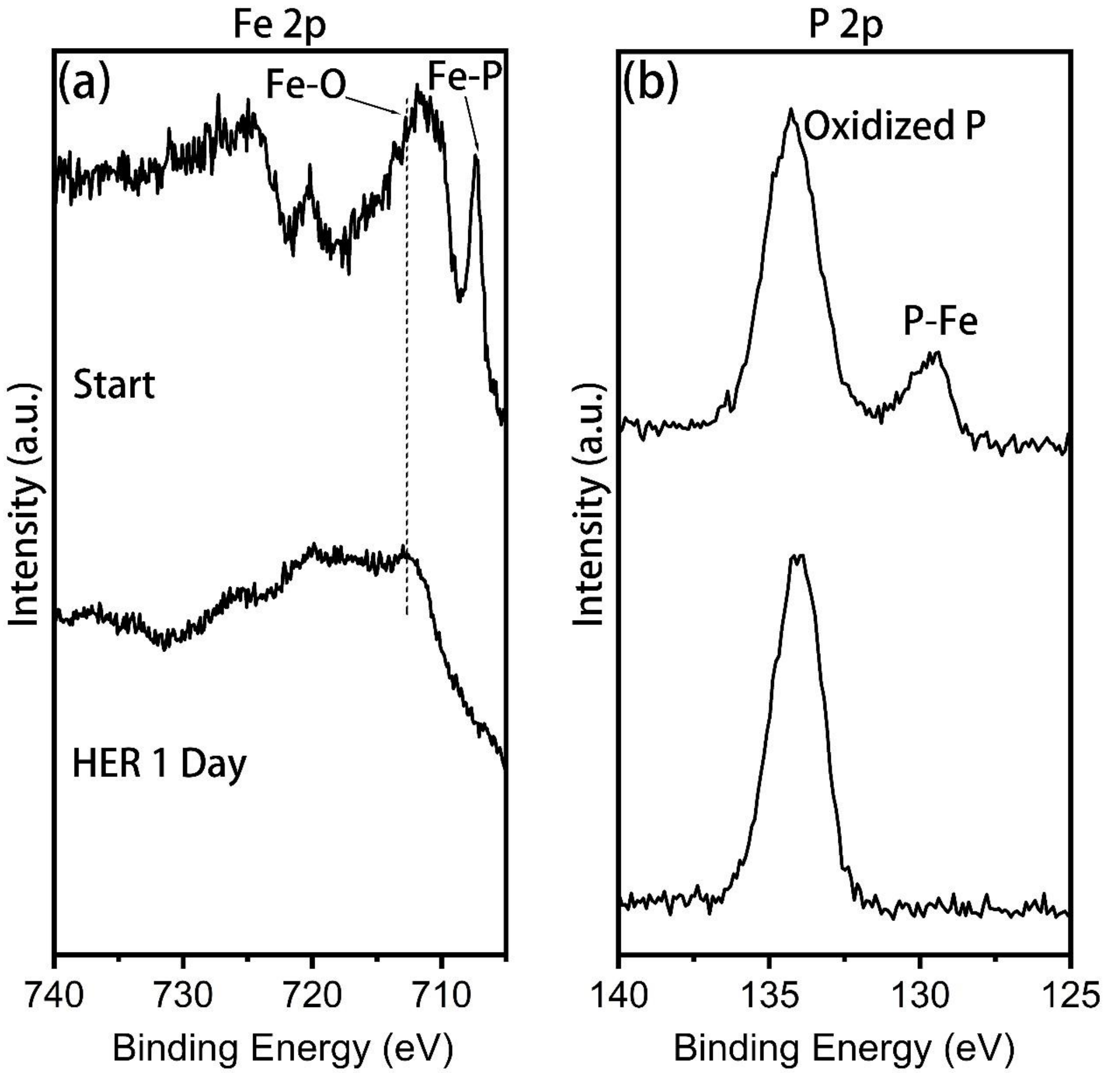

Figure S12. XPS Spectra of (a) Fe 2p and (b) P 2p for FeP/CNT before and after HER test at 10 mA cm^{-2} for 1 day.

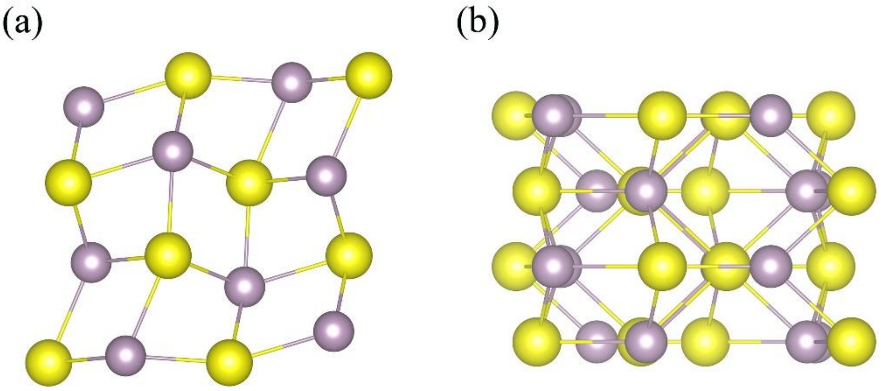

Figure S13. (a) Side view and (b) top view of FeP (110) slab, (yellow ball, Fe; grey ball, P).

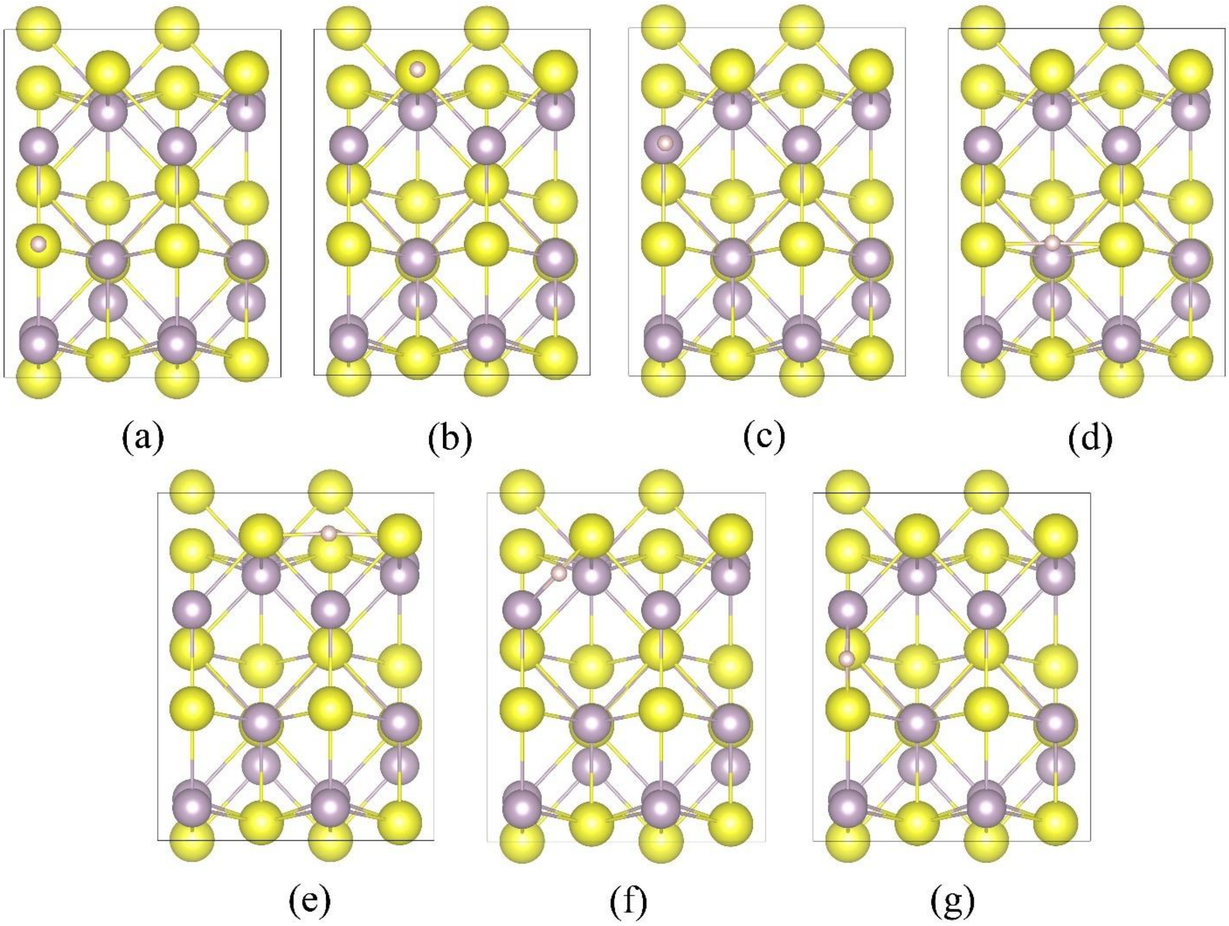

Figure S14. H adsorbed on (a) Fe_1, (b) Fe_2, (c) P, (d) short Fe-Fe bridge (S-Fe-Fe), (e) long Fe-Fe bridge (L-Fe-Fe), (f) P-Fe_2 bridge, and (g) P-Fe_1 bridge (yellow ball, Fe; grey ball, P, white ball, H).

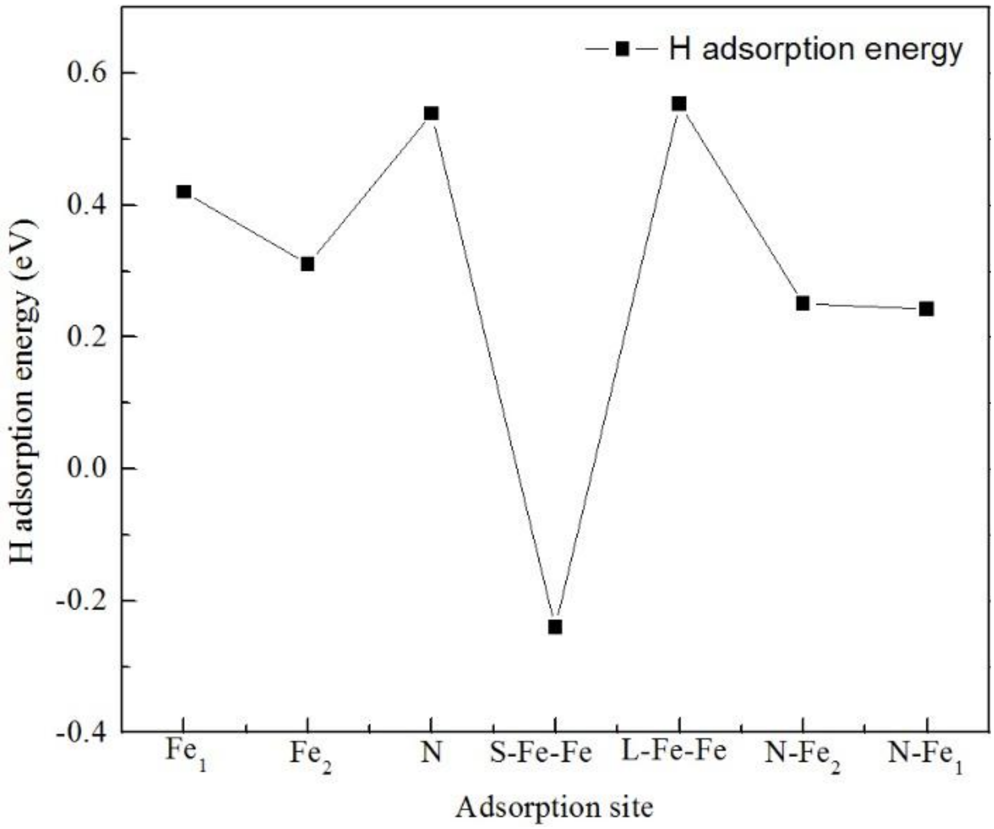

Figure S15. H adsorption energy for adsorption sites of (a) Fe1, (b) Fe2, (c) N, (d) short Fe-Fe bridge (S-Fe-Fe), (e) long Fe-Fe bridge (L-Fe-Fe), (f) N-Fe2 bridge, and (g) N-Fe1 bridge.

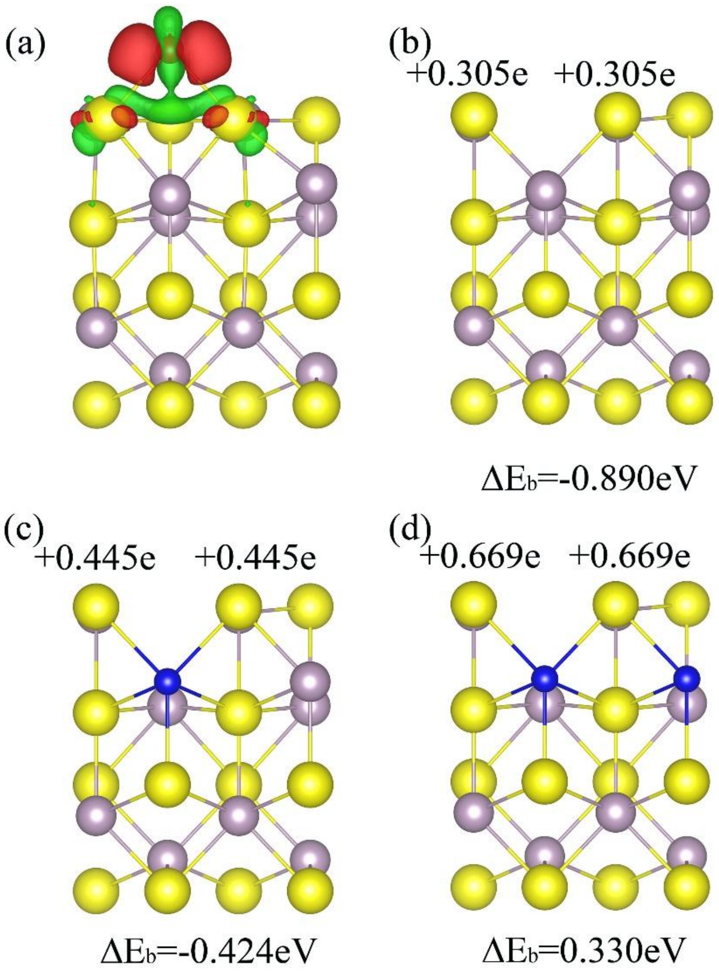

Figure S16. (a) Charge density difference of hydroxyl adsorbed on FeP. The charge depletion and accumulation were depicted by green and red, respectively. The Bader effective charges on Fe and corresponding binding energy of hydroxyl on Fe atom for (b) pristine FeP, (c) FeP doped with 1N and (d) FeP doped with 2N atoms(yellow ball, Fe; grey ball, P; red ball, O; blue ball, N; white ball, H).

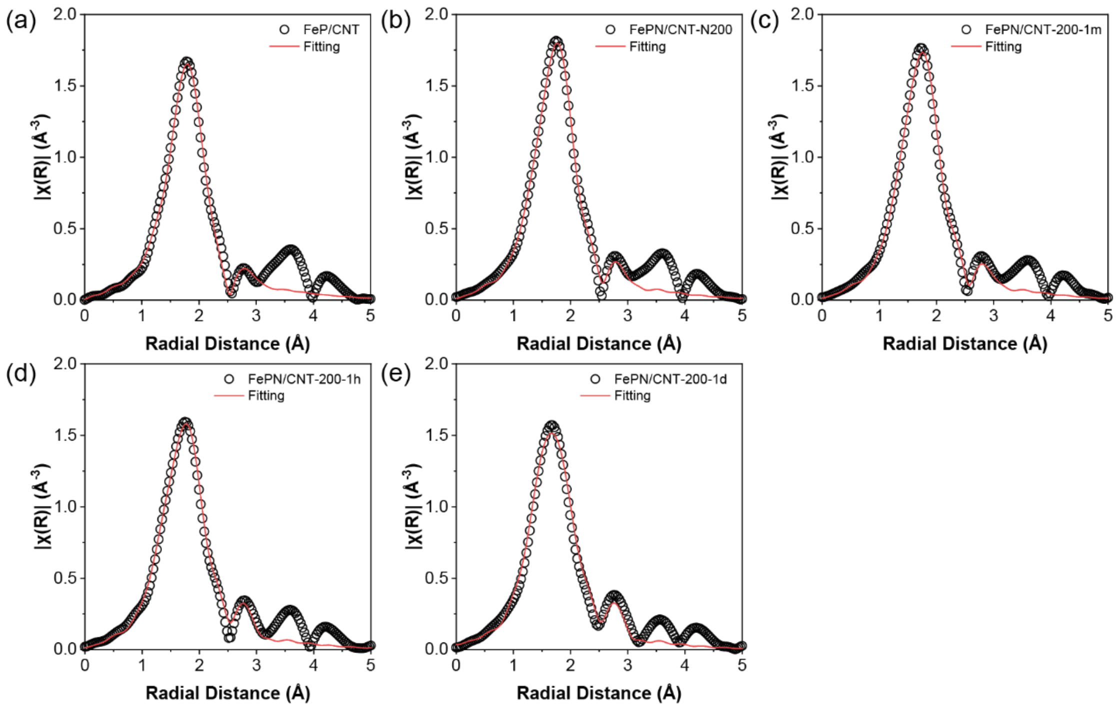

Figure S17. Fitting results of Fe K-edge k^2-weighted EXAFS spectra of (a) FeP/CNT, (b) FePN/CNT-200, (c) FePN/CNT-200-1m, (d) FePN/CNT-200-1h and (e) FePN/CNT-200-1d. The raw data points are plotted as circle dots and the fitted curve is shown in red. The fitting parameters are listed in Table S4.

Table S1. The surface ratio of Fe:P:N of FePN/CNT-200, FePN/CNT-250 and FePN/CNT-300 obtained by quantitative XPS analysis.

Sample	Ratio (Fe:P:N)
FePN/CNT-200	1:0.67:0.85
FePN/CNT-250	1:0.75:1.02
FePN/CNT-300	1:0.73:0.68

Table S2. Comparison of performance for HER electrocatalysts under neutral conditions

Electrocatalysts	Electrolytes	η_{10} (mV)	η_{50} (mV)	Tafel Slope (mV dec^{-1})	Ref
**FePN/CNT**	1M PBS	158	239	87	This paper
**SiO_2/PPy NTs-CFs**	1M PBS	~190	~290	100.2	17
**FeP/NPs@NPC**	1M PBS	386	>500	136	18
**Mn-NiO-Ni/Ni-F**	1M PBS	80	~250	121	19
**Karst NF**	1M PBS	110	~240	99	20
**MoP700**	1M PBS	196	N/A	79	21
**Ni_3Se_4/Ni foam**	1M PBS	~180	282	101	22
**MoP NA/CC**	1M PBS	187	~300	94	23
**NPNNS**	Seawater	144	>300	108	24
**CoO/$CoSe_2$**	0.5M PBS	337	434	131	25
**$Ni_{0.1}Co_{0.9}P$**	1M PBS	125	N/A	103	26
**Ni-Co-S-3/FTO**	1M PBS	280	N/A	93	27
**Fe@N-CNT/IF**	0.5M Na_2SO_4	525	N/A	199.6	28
**Co-B**	0.5M PBS	251	N/A	75	29
**U-CNT-900**	1M PBS	240	N/A	159	30
**NiS_x-3**	0.5M Na_2SO_4	221	N/A	101	31

Table S3. The process of HER in alkaline media.[32]

Step of HER in alkaline solution	Tafel slope in theory (mV/dec)
Volmer $H_2O + e^- = H^* + OH^-$	120
Heyrovsky $H_2O + e^- + H^* \rightarrow H_2 + OH^-$	40
Tafel $H^* + H^* = H_2$	30

Table S4. EXAFS fitting results of samples.

Scattering Paths	ΔE_0	Fe-P(1)			Fe-P(2)			Fe-Fe(1)			Fe-Fe(2)			Fe-Fe(3)			Fe-N/O		
**Sample**	**[eV]**	**C.N.**	**R [Å]**	σ^2	**C.N.**	**R [Å]**	σ^2	**C.N.**	**R [Å]**	σ^2	**C.N.**	**R [Å]**	σ^2	**C.N.**	**R [Å]**	σ^2	**C.N.**	**R [Å]**	σ^2
**FeP/CNT**	3.0±0.4	2.9±0.1	2.20±0.02	0.006±0.002	2.9±0.1	2.33±0.02	0.006±0.002	2.0±0.4	2.61±0.02	0.015±0.003	2.0±0.4	2.74±0.02	0.015±0.003	2.0±0.4	3.05±0.02	0.015±0.003	N/A	N/A	N/A
**FePN/CNT-200**	3.6±0.7	2.5±0.2	2.19±0.02	0.004±0.001	2.5±0.2	2.32±0.02	0.004±0.001	2.0±0.4	2.62±0.02	0.0120±0.003	2.0±0.4	2.75±0.02	0.0120±0.003	2.0±0.4	3.06±0.02	0.0120±0.003	1.0±0.4	1.92±0.03	0.004±0.001
**FePN/CNT-200-1m**	3.7±0.8	2.4±0.2	2.19±0.02	0.004±0.001	2.4±0.2	2.32±0.02	0.004±0.001	2.0±0.4	2.64±0.02	0.0120±0.003	2.0±0.4	2.77±0.02	0.0120±0.003	2.0±0.4	3.07±0.02	0.0120±0.003	1.2±0.5	1.93±0.03	0.004±0.001
**FePN/CNT-200-1h**	3.9±1.1	2.1±0.3	2.19±0.02	0.004±0.001	2.1±0.3	2.32±0.02	0.004±0.001	2.0±0.4	2.62±0.02	0.0120±0.003	2.0±0.4	2.75±0.02	0.0120±0.003	2.0±0.4	3.06±0.02	0.0120±0.003	1.4±0.5	1.91±0.03	0.004±0.001
**FePN/CNT-200-1d**	4.4±1.8	1.8±0.4	2.19±0.02	0.004±0.001	1.8±0.4	2.32±0.02	0.004±0.001	2.0±0.4	2.63±0.02	0.0120±0.003	2.0±0.4	2.76±0.02	0.0120±0.003	2.0±0.4	3.06±0.02	0.0120±0.003	2.2±0.9	1.92±0.03	0.004±0.001

Reference

[1] G. He, X. Han, B. Moss, Z. Weng, S. Gadipelli, F. Lai, A. G. Kafizas, D. J. L. Brett, Z. X. Guo, H. Wang, I. P. Parkin, *Energy Storage Mater.* **2018**, *15*, 380–387.

[1b] J. O. Hwang, J. S. Park, D. S. Choi, J. Y. Kim, S. H. Lee, K. E. Lee, Y. Kim, M. H. Song, S. Yoo, S. O. Kim, *ACS Nano* **2012**, *6*, 159-167.

[2] S. Zhao, J. Berry-Gair, W. Li, G. Guan, M. Yang, J. Li, F. Lai, F. Corà, K. Holt, D. J. L. Brett, G. He, I. P. Parkin, *Adv. Sci.* **2020**, *7*, 1903604.

[3] A. J. Dent, G. Cibin, S. Ramos, A. D. Smith, S. M. Scott, L. Varandas, M. R. Pearson, N. A. Krumpa, C. P. Jones, P. E. Robbins, *Journal of Physics: Conference Series* **2009**, *190*, 012039-012042.

[4] A. J. Dent, G. Cibin, S. Ramos, S. A. Parry, D. Gianolio, A. D. Smith, S. M. Scott, L. Varandas, S. Patel, M. R. Pearson, L. Hudson, N. A. Krumpa, A. S. Marsch, P. E. Robbins, *Journal of Physics: Conference Series* **2013**, *430*, 012023-012029.

[5] B. Ravel, M. Newville, *J. Synchrotron Rad.* **2005**, *12*, 537-541.

[6] C. H. Chu, C. W. Leung, *Integr. Equations Oper. Theory* **2001**, *40*, 391–402.

[7] R. A. Vargas-Hernández, *J. Phys. Chem. A* **2020**, *124*, 4053–4061.

[8] G. Kresse, J. Furthmüller, *Comput. Mater. Sci.* **1996**, *6*, 15–50.

[9] G. Kresse, J. Hafner, *Phys. Rev. B* **1994**, *49*, 14253-14269.

[10] P. E. Blöchl, *Phys. Rev. B* **1994**, *50*, 17953–17979.

[11] J. P. Perdew, K. Burke, M. Ernzerhof, *Phys. Rev. Lett.* **1996**, *77*, 3865–3868.

[12] K. Hu, M. Wu, S. Hinokuma, T. Ohto, M. Wakisaka, J. I. Fujita, Y. Ito, *J. Mater. Chem. A* **2019**, *7*, 2156–2164.

[13] J. K. Nørskov, J. Rossmeisl, A. Logadottir, L. Lindqvist, J. R. Kitchin, T. Bligaard, H. Jónsson, *J. Phys. Chem. B* **2004**, *108*, 17886–17892.

[14] G. L. Chai, Z. Hou, D. J. Shu, T. Ikeda, K. Terakura, *J. Am. Chem. Soc.* **2014**, *136*, 13629–13640.

[15] G. L. Chai, K. Qiu, M. Qiao, M. M. Titirici, C. Shang, Z. Guo, *Energy Environ. Sci.* **2017**, *10*, 1186–1195.

[16] G. L. Chai, Z. X. Guo, *Chem. Sci.* **2016**, *7*, 1268–1275.

[17] J.-X. Feng, H. Xu, S.-H. Ye, G. Ouyang, Y.-X. Tong, G.-R. Li, *Angew. Chemie.* **2017**, *129*, 8232–8236.

[18] Z. Pu, I. S. Amiinu, C. Zhang, M. Wang, Z. Kou, S. Mu, *Nanoscale* **2017**, *9*, 3555-3560.

[19] X. Lu, J. Pan, E. Lovell, T. H. Tan, Y. H. Ng, R. Amal, *Energy Environ. Sci.* **2018**, *11*, 1898–1910.

[20] X. Gao, Y. Chen, T. Sun, J. Huang, W. Zhang, Q. Wang, R. Cao, *Energy Environ. Sci.* **2020**, *13*, 174–182.

[21] X. Xie, M. Song, L. Wang, M. H. Engelhard, L. Luo, A. Miller, Y. Zhang, L. Du, H. Pan, Z. Nie, Y. Chu, L. Estevez, Z. Wei, *ACS Catal.* **2019**, *9*, 8712-8718.

[22] S. Anantharaj, J. Kennedy, S. Kundu, *ACS Appl. Mater. Interfaces* **2017**, *9*, 8714–8728.

[23] Z. Pu, S. Wei, Z. Chen, S. Mu, *Appl. Catal. B Environ.* **2016**, *196*, 193–198.

[24] Y. Huang, L. Hu, R. Liu, Y. Hu, T. Xiong, W. Qiu, M. S. (Jie T. Balogun, A. Pan, Y. Tong, *Appl. Catal. B Environ.* **2019**, *251*, 181–194.

[25] K. Li, J. Zhang, R. Wu, Y. Yu, B. Zhang, *Adv. Sci.* **2015**, *3*, 1500426.

[26] R. Wu, B. Xiao, Q. Gao, Y. Zheng, X. Zheng, J. Zhu, M. Gao, S. Yu, *Angew. Chemie* **2018**, *130*, 15671–15675.

[27] A. Irshad, N. Munichandraiah, *ACS Appl. Mater. Interfaces* **2017**, *9*, 19746–19755.

[28] J. Yu, G. Li, H. Liu, L. Zeng, L. Zhao, J. Jia, M. Zhang, W. Zhou, H. Liu, Y. Hu, *Adv. Sci.* **2019**, *6*, 1901458

[29] S. Gupta, N. Patel, A. Miotello, D. C. Kothari, *J. Power Sources* **2015**, *279*, 620–625.

[30] S. Gao, G. D. Li, Y. Liu, H. Chen, L. L. Feng, Y. Wang, M. Yang, D. Wang, S. Wang, X. Zou, *Nanoscale* **2015**, *7*, 2306–2316.

[31] Z. Qin, Y. Chen, Z. Huang, J. Su, Z. Diao, L. Guo, *J. Phys. Chem. C* **2016**, *120*, 14581–14589.

[32] H. Jiang, S. Zhao, W. Li, T. P. Neville, I. Akpinar, P. R. Shearing, D. J. L. Brett, G. He, *Green Energy Environ.* **2020,** in press.
